# Deciphering the molecular basis of maize cold tolerance via integrative regulatory network analysis

**DOI:** 10.1111/tpj.71197

**Published:** 2026-10-29

**Authors:** Tianshuo Li, Wei Wang, Chuang Jiang, Jinkui Cheng, Fei Yi, Liusheng Duan

**Affiliations:** ^1^ State Key Laboratory of Plant Environmental Resilience, Engineering Research Center of Plant Growth Regulator, Ministry of Education & College of Agronomy and Biotechnology China Agricultural University No.2 Yuanmingyuan West Road, Haidian Beijing China; ^2^ Center for Crop Functional Genomics and Molecular Breeding China Agricultural University Beijing China; ^3^ College of Agronomy and Biotechnology China Agricultural University Beijing China

**Keywords:** maize, cold stress, coronatine, hormone, RNA‐seq

## Abstract

Maize (*Zea mays* L.) is a globally critical but cold‐sensitive crop. Understanding the temporal organization of cold‐responsive, circadian‐associated, and hormone‐associated transcriptional programs is essential for deciphering how plants balance growth and survival under cold stress to ensure food security. We performed high‐resolution time‐series RNA‐seq on 108 maize seedling samples across 14 time points (0–36 h cold stress and 24 h recovery). Coronatine (COR) was utilized as a defined JA‐associated chemical perturbation to probe hormone‐associated transcriptional responses during cold stress. We identified 12 986 cold‐responsive and 2259 recovery‐responsive genes, revealing a recovery‐associated transcriptional transition characterized by suppressed photosynthesis‐related processes and enrichment of genes associated with lipid catabolism, accompanied by transient physiological stress during early recovery. Cold stress induced widespread phase shifts and reduced oscillation amplitudes among circadian‐associated transcripts, while COR identified distinct hormone‐associated transcriptional modules and candidate network‐associated genes, including *ZmWRKY71* and *ZmHSP28*, whose roles were further supported by molecular and genetic analyses. This study establishes a temporally resolved transcriptional framework for maize cold stress and recovery, integrating transcriptional dynamics, physiological responses, and experimentally supported regulatory nodes.

## Significance Statement

By integrating high‐resolution transcriptomics with chemical perturbation, this study identifies a recovery‐associated transcriptional transition accompanied by a transient physiological stress state during early recovery and a hormone‐associated transcriptional framework related to maize cold resilience. This framework provides insights into how plants balance growth and survival through phased transcriptional responses, offering potential targets for improving cold tolerance in crops.

## INTRODUCTION

Maize (*Zea mays* L.) is one of the most important crops globally, playing a critical role in ensuring world food security and supplying industrial raw materials (Erenstein et al., 2022; Shiferaw et al., 2011). However, as a C_4_ plant originating from tropical regions, maize is highly sensitive to low temperatures (Long & Spence, 2013; Schapendonk et al., 1989). With intensifying global climate change and the increasing frequency of extreme weather events, cold stress has emerged as a major abiotic constraint on maize production (Bailey‐Serres et al., 2019; Lesk et al., 2016). Particularly in high‐latitude regions such as Northeast China, which is the country's primary maize‐producing belt, frequent late‐spring cold spells often lead to soil temperatures remaining below the optimal threshold for germination (Guga et al., 2025; Liu et al., 2013). These conditions can delay seedling emergence by 10 to 15 days and trigger severe leaf chlorosis and root growth inhibition, potentially resulting in grain yield reductions of 20% to 30% in years with severe cold stress (Jiang et al., 2023; Song et al., 2020; Zhang et al., 2022). Such environmental constraints not only destabilize regional food supplies but also limit the adoption of high‐yielding, long‐growth‐duration varieties. Therefore, deciphering the molecular mechanisms underlying maize cold stress response to provide theoretical foundations and genetic targets for breeding cold‐tolerant varieties has become a critical scientific priority (Ding et al., 2024; Shi et al., 2018).

At the molecular level, plants have evolved intricate and multi‐layered signaling networks to perceive and adapt to the thermodynamic constraints imposed by cold stress. Cold perception is initiated by alterations in membrane fluidity, particularly membrane rigidification (Sangwan et al., 2001), which serve as primary physical cues. These changes activate membrane‐localized sensors, such as *COLD1*, that rapidly induce a characteristic cytosolic Ca^2+^ influx (Ma et al., 2015; Yuan et al., 2018). The transient Ca^2+^ signature is subsequently decoded by Calcium‐Dependent Protein Kinases (CDPKs) and Mitogen‐activated Protein Kinase (MAPK) cascades, notably the MEKK1‐MKK2‐MPK4 and MKKK‐MKK‐MPK3/6 modules, which act as central signal transducers (Teige et al., 2004; Zhao et al., 2017). These kinase cascades phosphorylate and activate key upstream regulators, including *ICE1* (Inducer of CBF Expression 1), thereby inducing the burst expression of the C‐Repeat Binding Factor (*CBF/DREB1*) family (Chinnusamy et al., 2003). As master transcription factors (TFs), CBFs activate downstream cold‐regulated genes containing CRT/DRE cis‐elements to synthesize cryoprotective proteins and osmolytes (Liu et al., 1998; Stockinger et al., 1997). Importantly, this canonical linear pathway does not operate in isolation but is modulated by the circadian clock. Core oscillator components, including *CIRCADIAN CLOCK ASSOCIATED 1* (*CCA1*) and *LATE ELONGATED HYPOCOTYL* (*LHY*), monitor these responses by binding directly to *CBF* promoters or physically interacting with signaling proteins (Dong et al., 2011; Kidokoro et al., 2021), ensuring that defense activation is synchronized with daily temperature cycles to optimize adaptation with minimal fitness costs. Although these interactions have been well established, particularly in Arabidopsis, most previous studies have focused on individual clock components or limited stress time points, and the temporal reorganization of circadian‐associated transcription during cold exposure and subsequent recovery remains insufficiently resolved in maize.

Beyond the foundational transcriptional machinery, phytohormones serve as critical endogenous messengers that orchestrate the balance between stress defense and growth. Among these, jasmonic acid (JA) signaling is intimately integrated into the cold response. Cold stress triggers JA accumulation and the subsequent CORONATINE INSENSITIVE 1 (COI1)‐mediated degradation of jasmonate ZIM‐domain (JAZ) repressors, which not only releases MYC TFs but also relieves the inhibition of ICE1, thereby potently activating downstream defense genes via the CBF‐dependent pathway (Hu et al., 2013). Counterbalancing this promotion, the ethylene (ET) signaling pathway employs its master TF ETHYLENE INSENSITIVE 3 (EIN3) to directly repress *CBF* transcription, establishing a negative feedback loop that prevents excessive defense responses from compromising plant growth (Shi et al., 2012). This intricate regulatory web is further interlaced with abscisic acid (ABA) and salicylic acid (SA) signaling; while ABA, a classical stress hormone, employs SUCROSE NON‐FERMENTING 1‐RELATED PROTEIN KINASE 2 (SnRK2) kinases to both independently activate protective genes and stabilize ICE1 proteins (Ding et al., 2015), SA modulates cellular redox homeostasis to indirectly maintain the stability of cold signaling components (Pokotylo et al., 2019). Together, these studies indicate extensive interactions among hormone‐associated pathways during plant cold responses. However, previous studies have generally focused on individual pathways or limited sampling points, leaving the temporal organization of hormone‐associated transcriptional responses during maize cold stress, particularly under a defined pathway perturbation, incompletely resolved.

To dissect such a complex regulatory web, chemical perturbation provides temporal resolution that is often difficult to achieve through conventional genetic approaches alone. Coronatine (COR), a polyketide toxin produced by bacteria, functions as a structural and functional mimic of the bioactive jasmonate‐L‐isoleucine (JA‐Ile) conjugate (Katsir et al., 2008). Unlike endogenous JA‐Ile, which is generated through plant jasmonate biosynthesis, COR is a bacterial phytotoxin that mimics JA‐Ile and is perceived through the same COI1‐JAZ receptor module; therefore, COR treatment does not itself indicate changes in endogenous JA biosynthesis or JA‐Ile accumulation. Exogenous COR application has been shown to enhance cold tolerance in several crops, including tomato and rice, by modulating antioxidant capacity and stress‐responsive gene expression (Feng et al., 2022; Liu et al., 2022). In this study, COR is employed primarily as a defined JA‐associated chemical perturbation to probe hormone‐associated transcriptional responses and thereby facilitate the temporal analysis of maize cold‐response pathways. By introducing a defined JA‐related perturbation, COR facilitates the identification and temporal comparison of transcriptional modules during cold adaptation.

Here, by integrating COR‐mediated perturbation with high‐resolution transcriptomic analysis, we systematically investigated the temporal organization of maize transcriptional responses during cold exposure and subsequent recovery. We established a comprehensive experimental system comprising four treatment regimes (normal control, cold stress, COR‐pretreated control, and COR‐pretreated cold stress) and generated deep transcriptomic data from 108 samples across a high‐density time course spanning 0–36 h of cold and 24 h of recovery (14 time points). This dataset enabled us to resolve phased cold‐ and recovery‐associated transcriptional responses and to characterize cold‐induced circadian‐associated transcriptional remodeling separately from COR‐associated hormone‐responsive programs. Building on this framework, we inferred candidate TF‐gene associations and experimentally tested selected regulatory relationships. EMSA and dual‐luciferase assays supported selected TF‐target interactions, while transgenic analyses identified *ZmWRKY71* and *ZmHSP28* as contributors to maize cold tolerance. Rather than defining new interactions among these established pathways, this work provides a temporally resolved framework for maize cold stress and recovery by integrating time‐resolved transcriptomics, physiological analyses, and functional validation.

## RESULTS

### Cold stress induces phased transcriptional responses in maize seedlings

To investigate the temporal dynamics of the maize seedling transcriptome under cold stress and to use COR as a defined chemical perturbation to probe associated transcriptional responses, we performed a high‐resolution time‐series RNA‐seq analysis. Based on a dose–response experiment, 0.5 μM COR was selected because it most effectively reduced cold‐induced leaf injury and ion leakage compared with 0, 0.1, and 1 μM COR (Figure S1a–c). The experimental design was as follows: twelve‐day‐old maize seedlings were first sprayed with 0 or 0.5 μM COR. After 24 h, the seedlings were subjected to differential temperature treatments, establishing four experimental regimes: normal control (25°C, N), cold stress (4°C, C), COR pretreatment under normal condition (NC), and COR pretreatment followed by cold stress (CC) (Figure 1a). The cold treatment lasted for 36 h, followed by a 24 h recovery period at 25°C. To capture the fine‐scale dynamics of transcriptional regulation, aerial tissues were collected at 0, 3, 6, 9, 12, 15, 18, 21, 24, and 36 h during the cold treatment, and at 6, 12, 18, and 24 h during the recovery phase. Two biological replicates were set up for each time point, generating a total of 108 independent samples for RNA‐seq. Phenotypic observations and quantitative assessment of injured leaf area showed that visible cold injury began to appear in the C group at 9 h, whereas its onset was delayed until approximately 18 h in the COR‐pretreated CC group (Figure 1b; Figure S1d).

**Figure 1:**
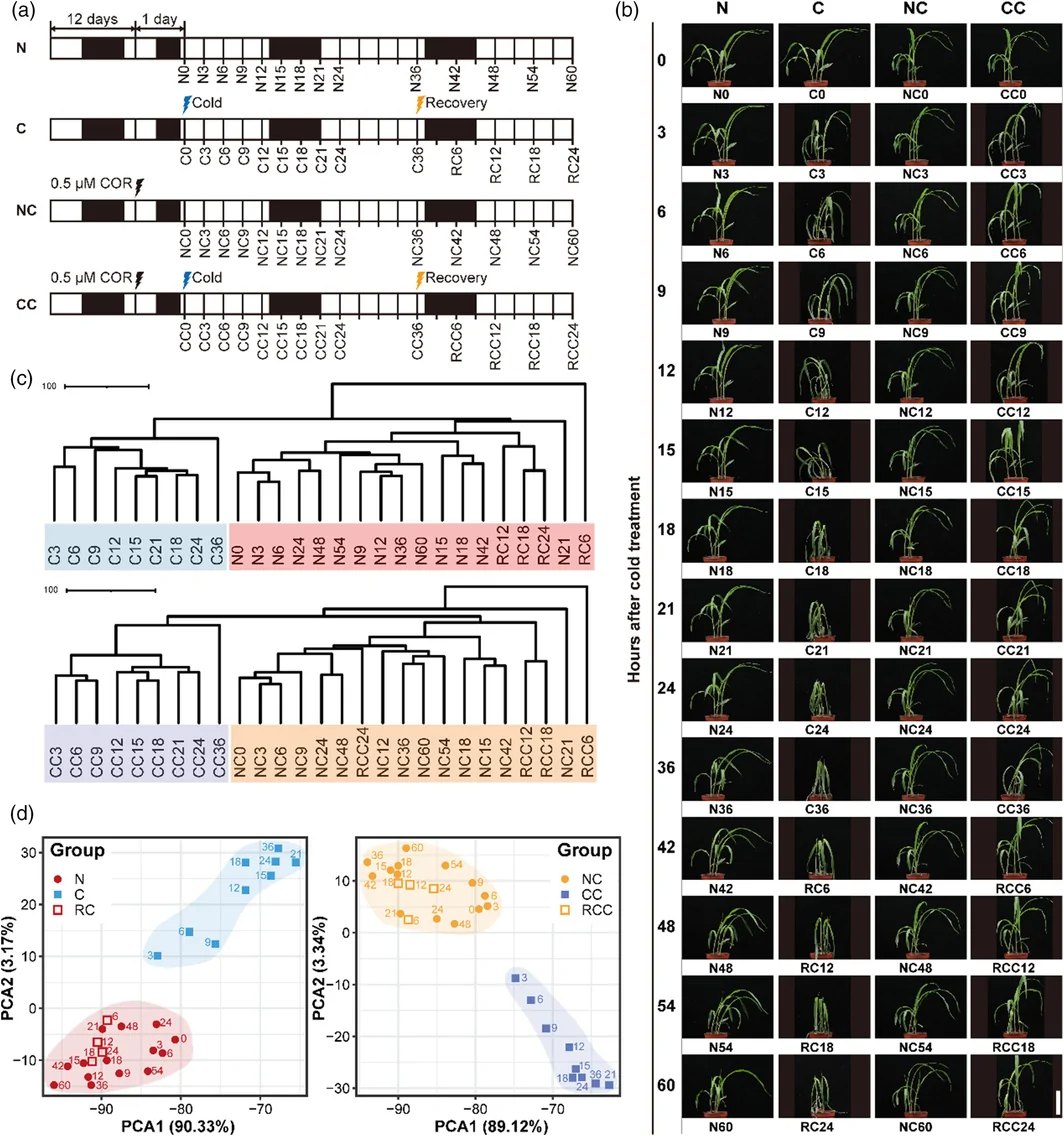
Experimental design, morphological changes, and transcriptome sample grouping of maize seedlings during cold stress and recovery. (a) Schematic overview of the experimental timeline. Maize seedlings were pre‐cultivated for 12 days, treated with 0 or 0.5 μM COR for 24 h, then exposed to 4°C cold stress for 36 h, followed by a 24 h recovery under normal conditions. Sampling time points during cold and recovery phases are indicated for each treatment group. N, normal control; C, cold stress; NC, 0.5 μM COR pretreatment under normal conditions; CC, 0.5 μM COR pretreatment under cold stress; RC and RCC represent the recovery stages following C and CC treatments, respectively. (b) Representative morphological phenotypes of maize seedlings under different treatments. Photographs show maize seedlings collected at indicated time points (0–60 h) under four treatments. Scale bar = 10 cm. (c) Hierarchical clustering analysis of transcriptome samples. The dendrogram shows the relationships among samples based on expression similarity. Background colors represent treatment groups: blue indicates C, red indicates N and RC, light purple indicates CC, and orange indicates NC and RCC. (d) Principal component analysis (PCA) of transcriptome samples. Each point represents one biological sample. Red circles represent N samples, blue filled squares represent C samples, red open squares represent RC samples, orange circles represent NC samples, light purple filled squares represent CC samples, and orange open squares represent RCC samples.

A total of 5.02 billion high‐quality reads were generated. On average, 96.59% of the reads were mapped to the B73 reference genome (RefGen_V4) (Jiao et al., 2017; Table S1), and then only uniquely mapped reads were used to calculate the normalized gene expression level as fragments per kilobase of transcript per million mapped reads (FPKM). Comparison of the two biological replicates showed that the expression values between them were highly correlated (average *R* ^2^ = 0.95). Hence, we took the average FPKM value of the two replicates as the expression level for the sample at each time point. In order to reduce the influence of transcription noise, we defined a gene as expressed only when the FPKM value of a gene was ≥1. Of the 108 samples, a total of 25 491 genes were expressed in at least one sample (Dataset S1).

To further evaluate the quality of the transcriptome dataset, we specifically examined the expression patterns of four well‐known cold‐responsive genes (Figure S3). The *DREB1s* family genes play central roles in plant cold responses (Jiang et al., 2022). Consistent with previous reports, *ZmDREB1.3* was strongly induced by cold stress in our data. Furthermore, cold signaling can also be transduced through the MAPK cascade, and our data corroborated this by the significant upregulation of *ZmMPK1* (an *AtMPK3* homolog) under cold stress (Li et al., 2017). Conversely, the peroxidase gene *ZmPRX108* and the aquaporin gene *ZmTIP4*;*3* (Sowinski et al., 2020; Zeng et al., 2024) were markedly downregulated after cold exposure (Figure S3). These results demonstrated that our data were trustworthy and appropriate for further analysis.

To obtain an overview of the relationships among the different transcriptomes, hierarchical clustering analysis and principal component analysis (PCA) were performed separately for the N versus C and NC versus CC comparisons. Hierarchical clustering analysis revealed consistent patterns in both experimental regimes: the cold‐stressed samples (C and CC) clustered into distinct clades, clearly separated from their respective normal conditions (N and NC), indicating that cold stress induced strong transcriptional signatures. Meanwhile, within the cold‐treated groups, samples were arranged in chronological order (from 3 h to 36 h), reflecting the progressive advancement of transcriptional reprogramming under cold stress (Figure 1c). The PCA results further corroborated these temporal patterns, where cold‐stressed samples (C and CC) were clearly distinguished from normal conditions (N and NC) along the first principal component and distributed continuously along the time trajectory in both comparisons. This parallel analysis demonstrated that the dynamic transcriptome remodeling during cold adaptation occurred in a similar trajectory for both the C and CC groups, separating distinctly from the N and NC groups, respectively (Figure 1d).

### Identification and temporal characterization of cold‐responsive genes in maize

To dynamically reveal the transcriptional changes induced by cold stress and decode the underlying regulatory networks, we identified cold‐responsive genes (CRGs) at each time point. Compared with the normal control (N) samples, a total of 12 986 CRGs, including 1048 TFs, were detected across the cold treatment time course. Based on *K*‐means clustering of their Norm‐normalized temporal expression profiles, the 12 986 CRGs were assigned to 18 clusters (M1‐M18) (Figure 2a and Dataset S2). Upregulated genes were assigned to clusters M1–M9, while downregulated genes were grouped into clusters M10–M18. Gene Ontology (GO) enrichment analysis indicated that upregulated clusters exhibited highly ordered functional transitions, whereas downregulated clusters were characterized by a persistent suppression of nucleic acid metabolism (Figure 2b).

**Figure 2:**
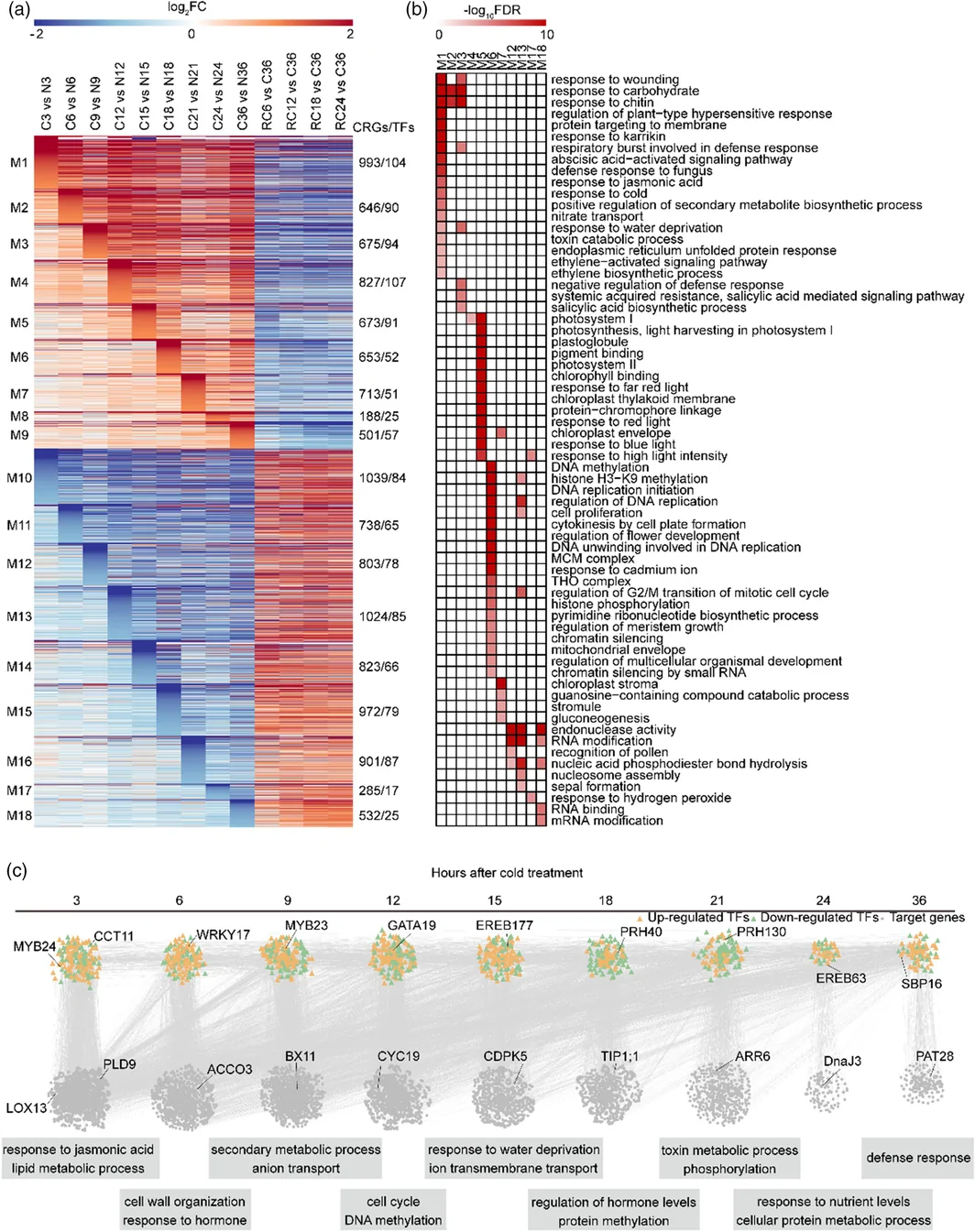
Temporal clustering, Gene Ontology enrichment, and time‐resolved TF‐gene association network analysis of maize cold‐responsive genes during cold stress. (a) *K*‐means clustering of CRGs based on their Norm‐normalized temporal expression profiles. The 12 986 CRGs were assigned to 18 clusters (M1–M18), which were subsequently designated according to the direction and onset time of their temporal expression changes. The heatmap displays expression changes of CRGs as log_2_ (Fold Change) (log_2_FC). Red indicates upregulated clusters (M1–M9), while blue indicates downregulated clusters (M10–M18). The numbers on the right denote the total number of genes and TFs in each cluster, respectively. (b) GO enrichment analysis of different CRG clusters. Significantly enriched GO terms (*Q* < 0.05) are shown for each cluster. (c) Time‐resolved TF‐gene association network of CRGs under cold stress. The network illustrates predicted TF‐gene associations across time points during cold treatment. Yellow and green triangles represent up‐ and downregulated TFs, respectively; gray circles represent candidate target genes, and gray lines indicate predicted TF‐gene association edges. Gray boxes below indicate the enriched biological processes associated with predicted target genes at each time point.

The rapidly activated Cluster M1 was predominantly enriched in hormone‐regulatory pathways, including “abscisic acid‐activated signaling pathway,” “response to jasmonic acid,” and “ethylene biosynthetic process.” Within this cluster, several core hub genes involved in signal transduction were identified. For instance, *ZmABH1*, a homolog of Arabidopsis *ABH1*, mediates ABA signaling by regulating transcript stability (Hugouvieux et al., 2001). Simultaneously, ZmABI28 and ZmABI33, belonging to the Abscisic Acid Insensitive (ABI) family, serve as central TFs in the ABA pathway (Finkelstein et al., 2002). Cluster M1 also contained some maize lipoxygenase genes, such as *ZmLOX1*, *ZmLOX4*, and *ZmLOX13*, indicating transcriptional changes associated with oxylipin metabolism during the early cold response (Ogunola et al., 2017). Furthermore, key ET signaling components such as ZmEIL8 and multiple ZmEREB (ETHYLENE‐RESPONSIVE ELEMENT BINDING PROTEIN) TFs were strongly induced, suggesting that hormone‐associated genes are involved in early transcriptional responses to cold stress (Yamaguchi‐Shinozaki & Shinozaki, 2006).

As the stress persisted, clusters M4, M5, and M6 showed transcriptional changes associated with photosynthetic and epigenetic processes. Although cold stress typically induces severe photoinhibition (Allen & Ort, 2001; Ensminger et al., 2006), clusters M4 and M5 were significantly enriched in photosynthesis‐related terms, including “photosystem I/II,” “light harvesting in photosystem I,” and “response to high light intensity,” indicating changes in photosynthesis‐related transcription during prolonged cold exposure. Additionally, “plastoglobule” was significantly enriched in these clusters. Cluster M6 was strongly enriched in “DNA methylation” and “histone H3‐K9 methylation,” indicating changes in epigenetic processes. Conversely, the downregulated Cluster M13 showed reduced expression of genes associated with nucleic acid‐related processes.

Finally, Cluster M7 showed significant enrichment in “chloroplast stroma”, “stromule,” and “gluconeogenesis,” indicating transcriptional changes associated with chloroplast organization and carbon metabolism. Meanwhile, the downregulated clusters M17 and M18 were enriched in genes associated with endonuclease activity.

Given that visible cold injury appeared at 9 h in the C group but was delayed to 18 h in the COR‐pretreated CC group, we further compared the cold‐responsive transcriptional changes at these two injury‐associated stages. This comparison was used to assess whether the transcriptional state at CC18 represented a simple temporal delay of that observed at C9 or a distinct response associated with COR pretreatment during prolonged cold exposure. To account for the effects of sampling time and COR pretreatment, differentially expressed genes (DEGs) were identified using the corresponding time‐ and treatment‐matched controls, namely C9 versus N9 and CC18 versus NC18. A total of 3217 and 6391 DEGs were identified in the two comparisons, respectively, of which 2135 were detected in both comparisons, whereas 1082 and 4256 were detected exclusively in C9 versus N9 and CC18 versus NC18, respectively (Figure S4). The 2135 shared DEGs accounted for 66.4% of the DEGs detected in C9 versus N9 but only 33.4% of those detected in CC18 versus NC18, indicating that the two injury‐associated stages retained a substantial common transcriptional component but differed markedly in their overall DEG composition. The DEGs detected in both comparisons were mainly enriched in transcriptional regulation, RNA biosynthetic processes, protein phosphorylation, and hormone‐mediated signaling, suggesting a shared core cold‐responsive program at the two injury‐associated stages (Figure S4b). In contrast, DEGs detected exclusively in C9 versus N9 were enriched in protein phosphorylation, peptide transport, RNA modification, and ion transmembrane transport (Figure S4c), a pattern consistent with rapid stress signaling and transport‐related adjustments accompanying the earlier onset of cold injury. By contrast, the much larger CC18‐specific DEG set was enriched in photosynthesis, DNA replication, cell cycle regulation, and metabolic processes (Figure S4d), indicating a broader transcriptional state involving photosynthesis‐, cell‐cycle‐, and metabolism‐related processes after prolonged cold exposure under COR pretreatment. These differences indicate that CC18 was not simply a time‐delayed replica of C9. Rather, the two injury‐associated stages shared a conserved core cold‐response program while exhibiting substantial differences in the additional transcriptional processes associated with injury onset. Thus, COR pretreatment was associated not only with delayed visible cold injury but also with a distinct transcriptional state during prolonged cold exposure.

To further resolve the regulatory architecture underlying these temporal transcriptional changes, we applied the context likelihood of relatedness (CLR) algorithm to construct a time‐resolved TF‐gene association network (Figure 2c). The inferred network contained 6760 genes, including 1048 TFs, and 13 011 predicted TF‐gene edges (Dataset S3). Network topology analysis revealed distinct shifts in functional modules corresponding to the gene expression patterns. Initially, predicted target genes of TFs were significantly enriched in “response to jasmonic acid” and “cell wall organization” indicating enrichment of early signaling‐related processes. Subsequently, the network architecture shifted toward modules related to “DNA methylation”, “cell cycle” and “ion transmembrane transport” followed by changes in these transcriptional processes. Ultimately, predicted target genes were enriched in “defense response” and “response to nutrient levels” indicating later enrichment of defense‐ and metabolism‐associated processes. Collectively, these findings illustrate dynamic transitions among hormone‐associated, photosynthesis‐related, and epigenetic transcriptional modules.

### Identification and functional characterization of recovery‐response genes following cold stress

To uncover transcriptional reprogramming events specific to the post‐stress recovery phase, we identified genes that were differentially expressed during recovery but not classified as CRGs during the cold phase, defining them as recovery‐response genes (RRGs). A total of 2259 RRGs were identified (Figure 3 and Dataset S4), representing transcriptional events unique to the transition from cold stress to normal growth. To characterize their temporal dynamics, RRGs were grouped into four distinct clusters using Mfuzz soft clustering (Figure 3a). Clusters R1 and R2 represented genes displaying maximal downregulation at 6 h and 12 h, respectively, whereas Clusters R3 and R4 characterized genes exhibiting peak upregulation at these corresponding time points.

**Figure 3:**
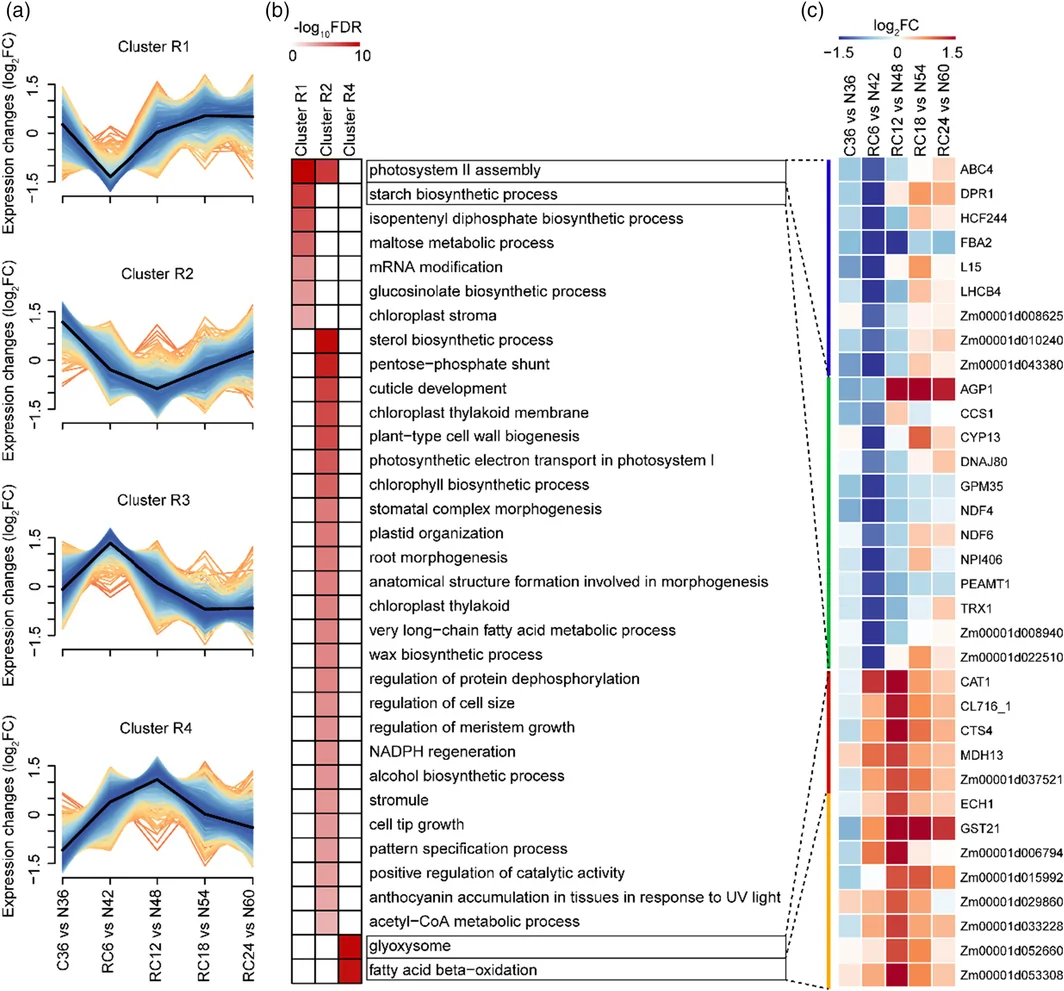
Expression clustering and functional enrichment of recovery‐response genes (RRGs). (a) Temporal expression clustering of recovery‐response genes. RRGs were clustered into four distinct expression patterns using the Mfuzz algorithm. Thin lines represent expression trajectories of individual genes, and thick lines indicate LOESS‐smoothed mean expression profiles for each cluster. (b) GO enrichment analysis of RRG clusters. Significantly enriched GO terms (*q* < 0.05) are shown for each cluster. The color intensity represents –log_10_(FDR), with darker colors indicating higher enrichment significance. (c) Expression profiles of representative recovery‐responsive genes. The heatmap illustrates the log_2_FC of representative recovery‐responsive genes at various recovery time points (RC versus N). The vertical color tracks on the left correspond to the specific functional categories identified in (b), as indicated by the dashed lines. The color scale ranges from blue (downregulated) to red (upregulated). Specific gene symbols are listed on the right.

GO enrichment analysis of the downregulated clusters (R1 and R2) revealed enrichment of genes associated with physiological processes involved in vegetative growth (Figure 3b). Specifically, genes in Cluster R1 (Figure 3c) were significantly enriched in “photosystem II (PSII) assembly” and “starch biosynthetic process”. This pattern indicates reduced expression of genes associated with photosynthetic and carbohydrate metabolism processes during early recovery. Notably, the PSII stability factor *ZmHCF244* (Li, Liu, et al., 2019) and the phosphoglycerate mutase‐encoding genes *ZmGPM35* (Zhao & Assmann, 2011) were specifically downregulated during this early recovery phase (Figure 3c). These transcriptional patterns indicate that early recovery is accompanied by reduced expression of genes associated with photosynthetic and primary carbohydrate metabolic processes.

The upregulated clusters (R3 and R4) were associated with distinct metabolic processes during recovery (Figure 3b). While Cluster R3 (peaking at 6 h) displayed a broad induction of diverse genes without significant pathway enrichment, Cluster R4 (peaking at 12 h) showed significant enrichment of catabolic pathways. Functions such as “glyoxysome” and “fatty acid beta‐oxidation” were significantly enriched (Figure 3b). Central to these metabolic shifts was the marked upregulation of key enzymatic components (Figure 3c), including the glyoxysomal determinant *ZmCTS4* (*Citrate Synthase 4*) (Eckardt, 2005) and the beta‐oxidation enzyme *ZmECH1* (*Enoyl‐CoA Hydratase 1*) (Fulda et al., 2002). The concurrent upregulation of ZmECH1 and ZmCTS4 was consistent with increased transcription of genes associated with fatty acid β‐oxidation and glyoxylate‐cycle processes during recovery.

Collectively, these findings indicate a recovery‐associated transcriptional transition within the first 12 h of recovery, characterized by downregulation of photosynthesis‐ and primary carbohydrate metabolism‐associated genes and enrichment of genes associated with glyoxysomal and fatty acid β‐oxidation processes. These transcriptional changes prompted us to examine whether the early recovery interval was accompanied by corresponding physiological alterations.

To determine whether the recovery‐associated transcriptional transition was accompanied by physiologically detectable changes, we further monitored chlorophyll‐related status, membrane integrity, oxidative stress, and carbohydrate dynamics during the recovery phase. After transfer from 4 to 25°C, SPAD values showed a transient decline at 6 h of recovery before gradually increasing, indicating delayed recovery of leaf chlorophyll status (Figure S5a). Meanwhile, ion leakage increased significantly at 6 h after recovery and subsequently decreased at later time points, suggesting transient membrane injury during early recovery (Figure S5b). Consistently, H_2_O_2_ accumulation peaked at 6 h of recovery, whereas MDA remained elevated during the early recovery period, indicating transient oxidative stress and lipid peroxidation during early recovery (Figure S5c,d). In addition, cold‐treated seedlings maintained higher soluble sugar levels and altered starch dynamics compared with normal controls throughout the recovery period (Figure S5e,f). Together, these physiological changes support the biological relevance of the recovery‐associated transcriptional transition and indicate that early recovery involves a transient stress state rather than an immediate return to normal growth conditions.

### Systematic identification of circadian‐associated genes and their temporal remodeling under cold stress

To characterize the temporal behavior of the maize circadian system under cold stress, we first identified rhythmic genes based on the time‐series transcriptome of samples under normal control (N). Rhythmic analysis was performed using the JTK_CYCLE algorithm implemented in the MetaCycle R package (Wu et al., 2016). Applying a Benjamini‐Hochberg adjusted *P*‐value (BH.Q) cutoff of 0.05 (Benjamini & Hochberg, 1995), we identified 2498 circadian genes exhibiting robust 24 h oscillations over the 0–24 h sampling period (3 h intervals) (Figure 4a and Dataset S5). The expression profiles of samples collected at 24 h intervals (e.g., N0, N24, N48, and N12, N36, N60) were highly consistent (Figure 4b), supporting the reproducibility of the identified rhythmic expression patterns under normal conditions and providing a reliable baseline for assessing cold‐associated temporal changes.

**Figure 4:**
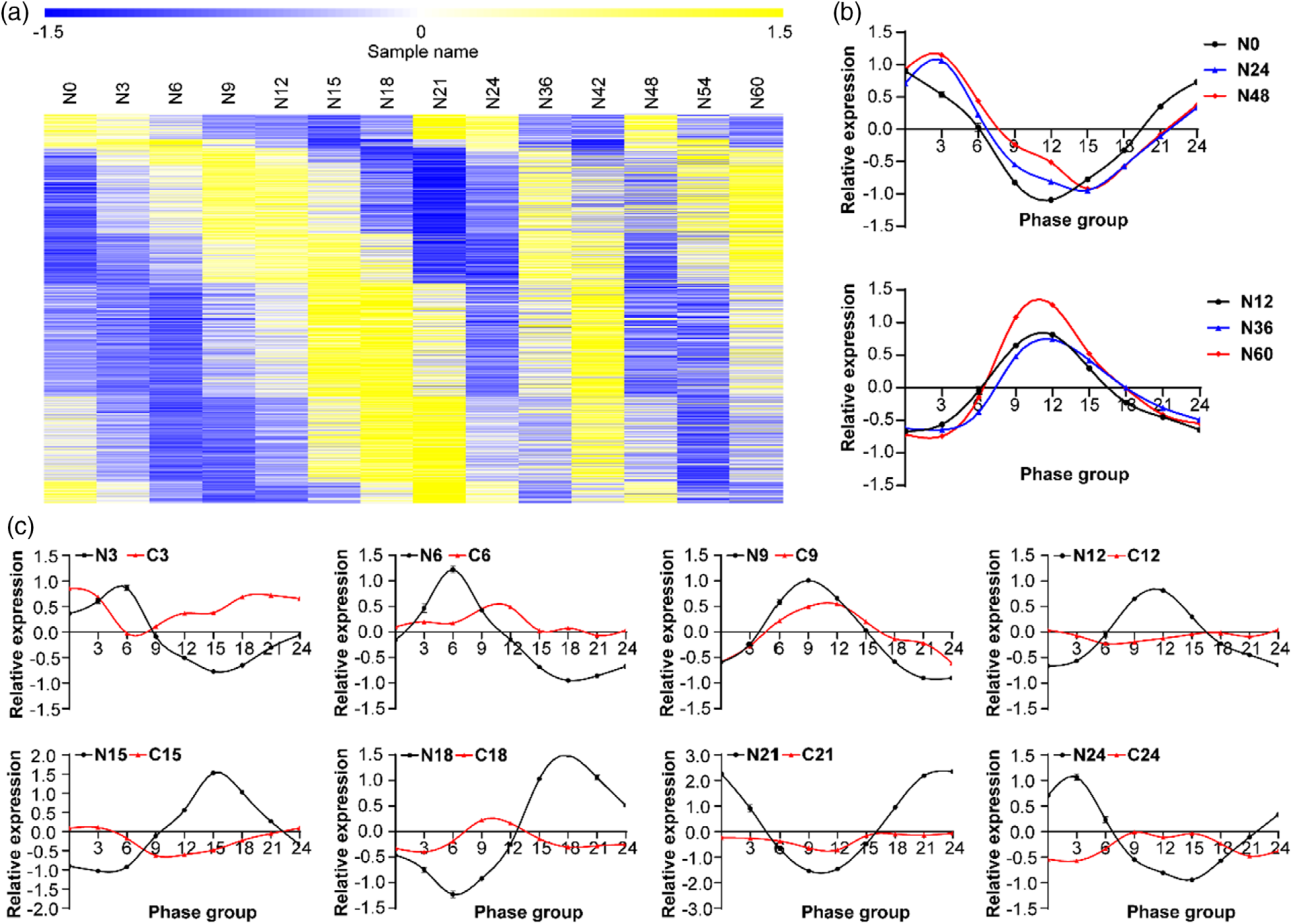
Circadian gene expression profiles in maize and their temporal remodeling under cold stress. (a) Heatmap of circadian gene expression profiles. The heatmap illustrates the expression profiles of identified circadian genes under normal control (N) conditions. Expression levels are represented as *Z*‐scores of FPKM values, and genes are arranged according to their phase (peak expression time). Yellow indicates high expression and blue indicates low expression. (b) Phase grouping of normalized circadian gene expression. Circadian genes were grouped according to their peak‐expression phase across the 0–24 h cycle at 3‐h intervals to display their rhythmic expression profiles. Data are shown as means ± SE (standard error). For some points, SE values are smaller than the symbol size and may not be visible. (c) Comparison of phase‐group expression patterns under normal control (N) and cold stress (C) conditions. Mean *Z*‐score expression profiles of genes assigned to each phase group were compared between N and C at 3, 6, 9, 12, 15, 18, 21, and 24 h. Each line plot represents the normalized phase‐group expression pattern at the indicated time point. N samples are shown in black and C samples in red. Data are presented as means ± SE, and SE values smaller than the symbol size may not be visible.

Following the onset of cold treatment, the phase of circadian genes exhibited a global delay of approximately 3–12 h. Specifically, peaks originally occurring at 6 h shifted to 12 h, and those at 9 h were delayed to 12 h under cold stress. Notably, by 12 h of cold treatment, phase clustering and reorganization were evident across multiple rhythmic groups, indicating substantial changes in the temporal organization of circadian‐associated transcription under cold stress (Figure 4c). These results reflect that cold stress alters the temporal expression patterns of circadian‐associated genes, characterized by phase shifts and reduced oscillation amplitudes. Simultaneously, we observed a marked attenuation in oscillation amplitude, resulting in reduced day‐night expression contrast, with the most pronounced damping occurring at 12 h of cold treatment. The circadian‐associated genes displayed phase reassignment or markedly reduced rhythmic expression patterns, implying that cold stress not only delayed peak timing but also reduced the apparent robustness of oscillatory expression (Figure S6). This cold‐induced remodeling of circadian‐associated expression, characterized by widespread phase delays and amplitude damping, may reflect temporal transcriptional adjustments under cold stress.

Given the close association between circadian regulation and abiotic stress responses, we compared the identified circadian gene set with the CRGs. A total of 1518 out of 2498 (60.8%) circadian genes overlapped with CRGs, representing 11.7% of all CRGs (Figure S7). Although circadian‐associated genes constituted a subset of the total cold‐responsive transcriptome, the high proportion of rhythmic genes that were also cold‐responsive indicates that cold stress affects a defined temporal layer of gene expression. Prominent among these overlapping genes were central circadian components, represented by *ZmGI1* and *ZmGI2* (*GIGANTEA*). *GI* is widely recognized as a pivotal node integrating circadian rhythms with abiotic stress responses, particularly in cold adaptation (Cao et al., 2005; Mishra & Panigrahi, 2015). Together, these findings indicate that cold stress is associated with substantial temporal remodeling of circadian‐associated transcription in maize seedlings.

### Core cold‐responsive genes show coordinated hormone‐associated and growth‐related transcriptional patterns

COR has been demonstrated to significantly enhance cold tolerance in tomato by inducing extensive transcriptional reprogramming and cold‐related epigenetic adaptations (Liu et al., 2022). To systematically dissect the key regulatory nodes governing this process in maize, we sought to identify genes at the intersection of cold signaling and COR modulation. We first identified 3597 COR‐responsive genes (COR‐RGs) defined as the union of differentially expressed genes responding to COR pretreatment under either normal (NC versus N) or cold stress (CC versus C) conditions (Dataset S6). To further pinpoint the COR‐modulated cold‐response components, we performed an overlap analysis between these COR‐RGs and the 12 986 identified CRGs. This intersection yielded a robust subset of 3013 genes, which were designated as “core CRGs” (Figure S8 and Dataset S7).

To explore their regulatory logic, the 3013 core CRGs were grouped into four clusters based on their expression trends (Figure 5a–d). Cluster I genes were induced by cold stress and further amplified by COR (N, NC < C < CC); Cluster II included cold‐induced genes whose expression was suppressed by COR (N, NC < C and CC < C); Cluster III comprised genes suppressed by cold but recovered or upregulated following COR treatment (N, NC > C and CC > C); and Cluster IV represented genes suppressed by both cold and COR (N, NC > C > CC). Notably, Clusters I and III showed distinct COR‐associated transcriptional patterns, as GO enrichment analysis revealed that both Clusters I and III were significantly associated with “response to jasmonic acid” and “secondary metabolic process” (Figure S9a,c). This convergent enrichment suggests two COR‐associated transcriptional response patterns: COR enhanced the expression of a subset of cold‐induced defense‐associated genes (Cluster I) while alleviating the suppression of another group of CRGs (Cluster III).

**Figure 5:**
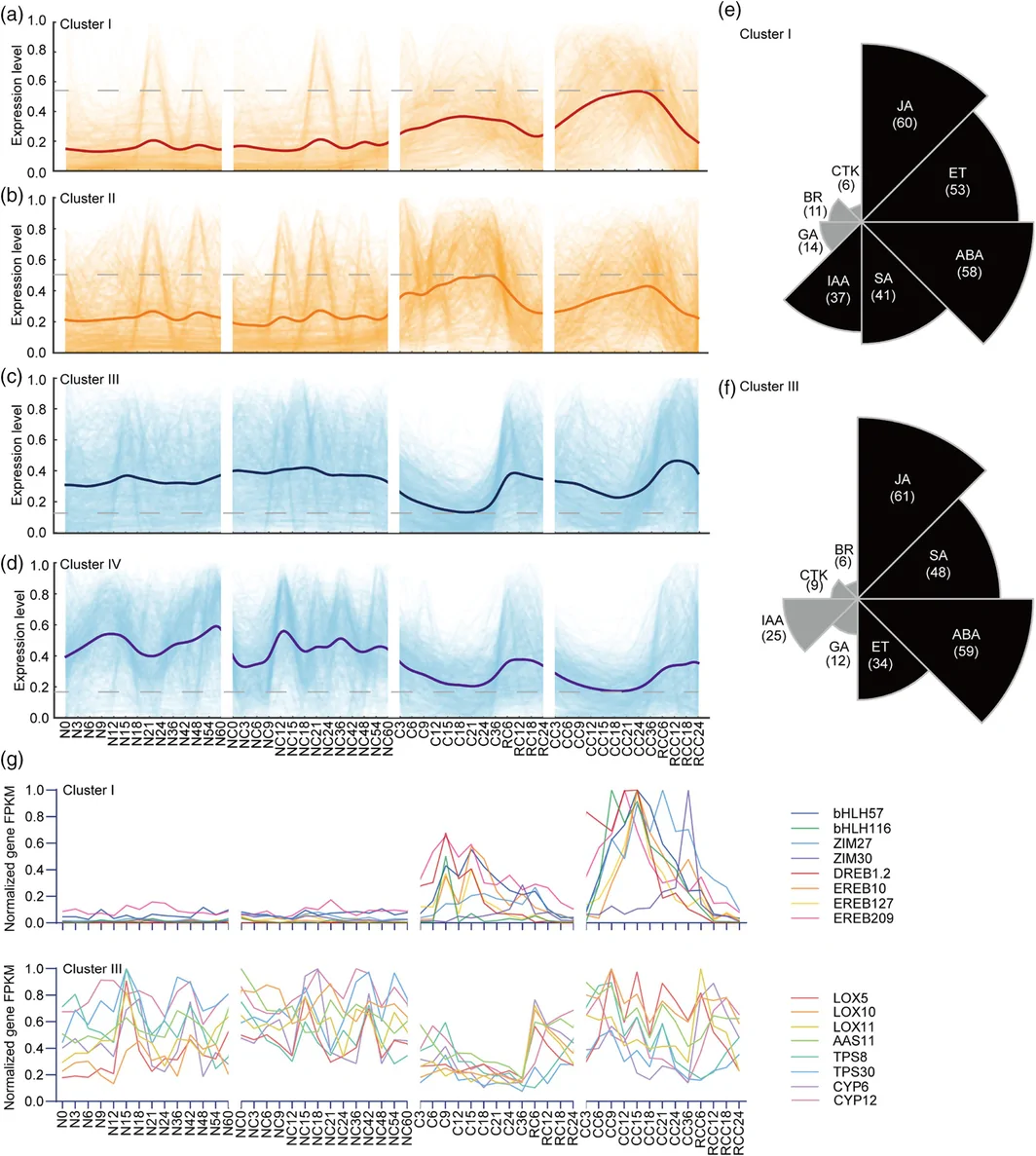
Clustering and hormone pathway analysis of core cold‐responsive genes (core‐CRGs). (a–d) Expression pattern clustering of core‐CRGs. Genes were grouped into four clusters (Cluster I‐IV) based on their expression profiles across four treatments (N, C, NC, and CC) during 0–60 h. Thin lines represent normalized expression trajectories of individual genes, and thick lines show LOESS‐smoothed mean expression patterns for each cluster (span = 0.5). Colors indicate different clusters: red (Cluster I), orange (Cluster II), dark blue (Cluster III), and purple (Cluster IV). (e, f) Hormone‐related pathway enrichment in Cluster I and Cluster III. Black sectors indicate significantly enriched pathways (*q* < 0.001), while gray sectors represent non‐significant pathways. Numbers denote the count of genes in each pathway. (g) Normalized expression levels of representative genes in Cluster I and Cluster III. The line plots illustrate the dynamic expression patterns (normalized FPKM) of selected genes across all treatment time points. Gene symbols are listed on the right.

Consistent with the GO enrichment results, pathway analysis further indicated that Clusters I and III contained enriched hormone‐associated genes, particularly those involving JA, ET, SA, and ABA signaling (Figure 5e,f). The distinct regulatory roles of Cluster I and III were most evident in the JA pathway (Figure 5g) where a distinct functional partition emerged: JA biosynthesis genes (*ZmLOX5*, *ZmLOX10*, and *ZmLOX11*) belonged to Cluster III, as COR pretreatment effectively counteracted their cold‐induced suppression; in contrast, signaling components including *ZmbHLH57*, *ZmbHLH116*, *ZmZIM27*, and *ZmZIM30* belonged to Cluster I, as their cold‐induced expression was further increased under COR pretreatment. A parallel regulatory architecture was observed for the ABA and SA pathways (Figure S10). Within Cluster I, COR treatment significantly enhanced the cold‐induced expression of key signaling transducers, including the kinases *ZmCDPK19* and *ZmCDPK32* (*CALCIUM‐DEPENDENT PROTEIN KINASE 19/32*), *ZmMPK7* (*MITOGEN‐ACTIVATED PROTEIN KINASE 7*), and multiple transcription factors such as *ZmWRKY15*, *ZmWRKY29*, and *ZmWRKY32*. Complementing these expression changes, Cluster III featured the COR‐mediated alleviation of cold‐induced suppression for genes associated with ABA/SA biosynthesis and homeostasis, such as *ZmSK1* (*SHIKIMATE KINASE 1*), *ZmUGT1* (*UDP‐GLYCOSYLTRANSFERASE 1*), *ZmGLL1* (*GLIP‐LIKE LIPASE 1*), and *ZmPAL6* (*PHENYLALANINE AMMONIA‐LYASE 6*). Several ET‐associated TFs in Cluster I showed enhanced cold‐induced expression under COR pretreatment. Multiple EREB TFs, including *ZmDREB1.2*, *ZmEREB10*, *ZmEREB127*, and *ZmEREB209*, followed this Cluster I‐like pattern of heightened induction in COR‐pretreated seedlings. Beyond these signaling modules, Cluster III also encompassed a diverse array of metabolic and defense‐related genes, including ZmAAS11 (AMINO ACID PERMEASE 11), ZmTPS8/30 (TERPENE SYNTHASE 8/30), and ZmCYP6/12 (CYTOCHROME P450 6/12), whose cold‐induced downregulation was significantly attenuated by COR.

Clusters II and IV showed additional COR‐associated transcriptional patterns. Genes in Cluster II, including lipid‐responsive components such as *ZmbZIP114* and *ZmCDPK9* (Figure S11), displayed cold‐induced expression that was dampened by COR pretreatment. Enriched in “response to lipid” (Figure S9b), this pattern may reflect an attenuated transcriptional response. In contrast, Cluster IV comprised genes that were consistently downregulated under both cold stress and COR treatment, exemplified by key cell cycle regulators such as *ZmCYC1*, *ZmCYC3*, and *ZmCYC4* (Figure S11). This cluster was significantly enriched in “regulation of DNA replication” and “cell cycle process” (Figure S9d). This indicates a broad downregulation of genes associated with growth and developmental processes under cold stress and COR pretreatment.

Collectively, these distinct transcriptional patterns, which were associated with JA, ET, SA, and ABA‐related responses (Clusters I and III) and with growth‐ and metabolism‐related processes (Clusters II and IV), constitute a transcriptional framework associated with maize cold stress responses.

### Hormone‐associated network identifies candidate TFs involved in the maize cold response

To further dissect the transcriptional regulatory architecture underlying maize cold tolerance, we inferred a hormone‐associated TF‐gene network (Figure 6a) by identifying up to three high‐connectivity candidate TFs from each of the four hormone signaling pathways (JA, ET, ABA, and SA) enriched in Clusters I and III (Dataset S8). The resulting network comprises 22 candidate TFs and 327 predicted target genes, forming a total of 357 predicted TF‐gene associations. To assess independent support for selected network predictions, we cross‐referenced the predicted TF‐gene associations with a recently published maize TF DAP‐seq dataset (Huo et al., 2025). Consistent with the network predictions, the presence of distinct DAP‐seq binding peaks for *ZmEREB2* in the promoter regions of *ZmDEK36* and *ZmPDC2*, as well as for *ZmWRKY27* in the promoter regions of *ZmHSP6* and *ZmNAC70* (Figure 6b), indicates that these predicted interactions are substantiated by physical binding, thereby supporting selected predictions of the reconstructed network.

**Figure 6:**
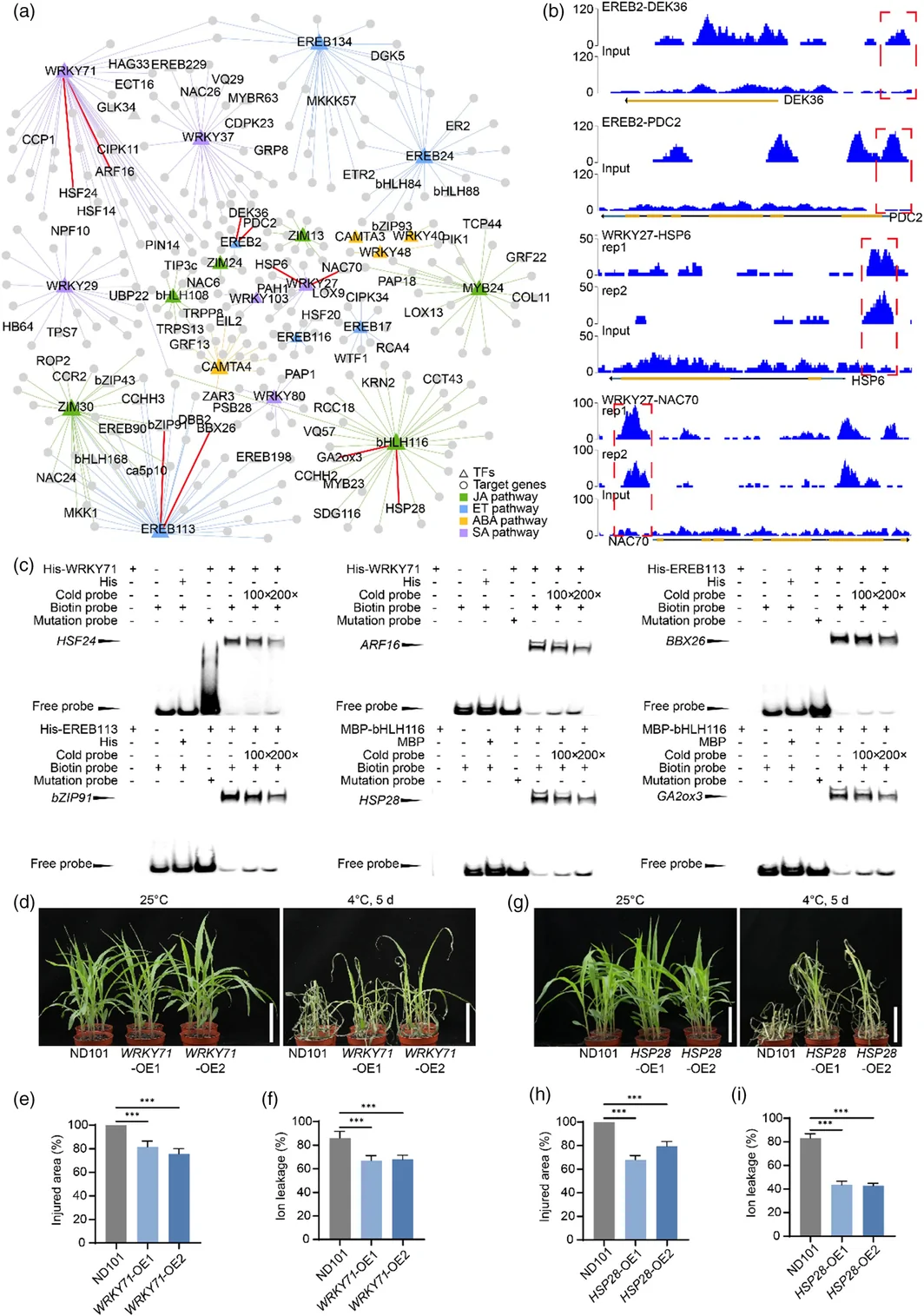
Hormone‐associated TF‐gene network and functional validation of selected network‐associated components under cold stress. (a) Inferred hormone‐associated TF‐gene network under cold stress. The network includes up to three high‐connectivity candidate TFs from each of the four hormone‐associated pathways (JA, ET, ABA, and SA) enriched in Clusters I and III. Triangles represent TFs, and circles represent predicted target genes. Green, blue, yellow, and purple nodes represent genes associated with the JA, ET, ABA, and SA pathways, respectively. Lines indicate predicted TF‐gene associations. Bold red lines highlight selected TF‐gene associations examined using DAP‐seq or EMSA in panels (b) and (c). (b) DAP‐seq profiles showing ZmEREB2 binding at the promoter regions of ZmDEK36 and ZmPDC2 and ZmWRKY27 binding at the promoter regions of ZmHSP6 and ZmNAC70. The blue peaks represent DNA fragments enriched by the respective TFs. Red dashed boxes highlight specific binding events in the promoter regions. The “Input” track serves as the negative control. (c) EMSA analysis of the binding of His‐ZmWRKY71 to the *ZmHSF24* and *ZmARF16* promoter probes, His‐ZmEREB113 to the *ZmBBX26* and *ZmbZIP91* promoter probes, and MBP‐ZmbHLH116 to the *ZmHSP28* and *ZmGA2ox3* promoter probes. Biotin‐labeled wild‐type probes and corresponding probes carrying mutations in the predicted cis‐elements were used. To verify binding specificity, excess unlabeled wild‐type competitor probes (100‐ and 200‐fold excess) were added to the reaction mixtures as competitors. The ‘+’ and ‘−’ signs indicate the presence or absence of the corresponding components. (d–f) Cold tolerance phenotypes and physiological indices of *ZmWRKY71*‐OE plants. (d) Representative phenotypes of *ZmWRKY71*‐OE lines and wild‐type (ND101) plants after 5 days of cold treatment (4°C). (e) Injured area of leaves and (f) ion leakage rate of *ZmWRKY71*‐OE lines under cold stress. (g–i) Cold tolerance phenotypes and physiological indices of *ZmHSP28*‐OE plants. (g) Representative phenotypes of *ZmHSP28*‐OE lines and wild‐type (ND101) plants after 5 days of cold treatment (4°C). (h) Injured area of leaves and (i) ion leakage rate of *ZmHSP28*‐OE lines under cold stress. In (d) and (g), scale bars: 10 cm. In (e), (f), (h), and (i), data are means ± SD from 3 biological replicates. The statistical significance was determined by a two‐sided *t*‐test (***P < 0.001).

Within this integrative network, the TFs exhibiting the highest degree of connectivity were *ZmWRKY71* (40 target genes), *ZmEREB113* (39), and *ZmbHLH116* (31) (Figure 6a). *ZmWRKY71* is a homolog of the Arabidopsis cold‐induced factor *AtWRKY34* (Zou et al., 2010) and was predicted to target the heat shock factor *ZmHSF24*, the auxin response factor *ZmARF16*, and the calcium‐sensing protein *ZmCIPK11*. *ZmEREB113*, a member of the AP2/ERF TF family (Xie et al., 2019), was predicted to target multiple hormone‐ and stress‐associated genes within the network, including the JA signaling repressor *ZmZIM30*, the lipoxygenase *ZmLOX13*, and stress‐responsive TFs such as *ZmBBX26* and *ZmbZIP91*. These predicted associations suggest that *ZmEREB113* serves as a candidate regulator connecting multiple hormone‐associated transcriptional programs during cold stress. *ZmbHLH116* exhibits regulatory features similar to those of the classic cold‐response master regulator *ICE1* (Ding et al., 2019) and was predicted to target the heat shock protein *ZmHSP28* and the GA catabolic enzyme *ZmGA2ox3*, suggesting a potential association with protein homeostasis‐ and GA metabolism‐related responses under cold stress.

To experimentally validate selected TF‐target interactions predicted by the hormone‐associated TF‐gene network, we performed EMSA to assess the direct binding of ZmWRKY71 to the promoters of *ZmHSF24* and *ZmARF16*, ZmEREB113 to the promoters of *ZmBBX26* and *ZmbZIP91*, and ZmbHLH116 to the promoters of *ZmHSP28* and *ZmGA2ox3*. Clear mobility‐shifted bands were observed when the purified TF fusion proteins were incubated with the corresponding biotin‐labeled promoter probes, whereas the His‐ or MBP‐tag controls produced no detectable shifts (Figure 6c). Mutation of the predicted cis‐elements abolished or markedly reduced the shifted bands, and increasing amounts of unlabeled competitor probes progressively weakened the binding signals, supporting the sequence‐specific binding of these TFs to their respective target promoters. Consistent with the EMSA results, dual‐luciferase reporter assays in maize protoplasts showed that ZmWRKY71, ZmEREB113, and ZmbHLH116 significantly repressed the activities of their corresponding target promoters (Figure S12).

Based on these confirmed physical interactions and their transcriptional responsiveness to cold stress (Figure S13), we selected the pivotal TF *ZmWRKY71* and the key functional gene *ZmHSP28* to further evaluate their contributions to cold tolerance. The coding sequences of *ZmWRKY71* and *ZmHSP28* were expressed under the control of the maize ubiquitin‐1 promoter in the pBECXUN vector (Figure S14a,b). Six independent *ZmWRKY71*‐overexpression events and two independent *ZmHSP28*‐overexpression events were obtained. Quantitative RT‐PCR confirmed significantly elevated transcript levels of *ZmWRKY71* and *ZmHSP28* in the corresponding transgenic lines, and two independent lines for each gene were used for subsequent analyses (Figure S14c,d). Moreover, following cold exposure, the transcript levels of the ZmWRKY71 target genes *ZmARF16* and *ZmHSF24* were generally lower in the *ZmWRKY71*‐OE lines than in ND101, particularly during 6–24 h of cold treatment, further supporting their negative regulation by *ZmWRKY71* under cold stress (Figure S14e,f). Phenotypic assays demonstrated that overexpression of *ZmWRKY71* and *ZmHSP28* markedly enhanced cold resistance in maize seedlings (Figure 6d–i). After exposure to 4°C for 5 days, the relative injured leaf area of *ZmWRKY71*‐OE and *ZmHSP28*‐OE plants was significantly lower than that of the wild type (ND101) (Figure 6e,h). Ion leakage analysis further revealed that cold‐induced plasma membrane damage was substantially mitigated in both overexpression lines (Figure 6f,i). Together, these results provide functional support for selected network‐associated components predicted by the network and show that *ZmWRKY71* and *ZmHSP28* contribute to maize cold tolerance.

## DISCUSSION

In this study, we generated a high‐resolution time‐series transcriptome map of maize seedlings under cold stress, covering a comprehensive trajectory from initial perception to the recovery phase. Comprehensive analysis depicted a phased transcriptional architecture and distinct responsive patterns during the recovery phase, revealing a sophisticated temporal coordination of the maize cold stress response. Through the systematic characterization of circadian‐associated transcriptional changes and the dissection of hormone‐related gene networks, we revealed widespread phase shifts in circadian‐associated expression patterns and identified the potential roles of *ZmWRKY71* and *ZmHSP28* in maize cold tolerance.

Cold stress is closely related to cellular signaling transduction and systemic transcriptional reprogramming (Shi et al., 2018). The induction of cold‐responsive transcriptional networks, integrated with changes in hormone‐associated expression programs, is consistent with phased responsive models involving cellular protection, hormone‐associated transcriptional changes, and metabolic adjustment (Ding & Yang, 2022; Hu et al., 2013). We further observed that during the subsequent recovery stage, genes associated with photosystem assembly and primary carbon metabolism were downregulated. This pattern indicates persistent transcriptional adjustments in photosynthesis‐ and carbon metabolism‐associated processes during recovery. The accompanying physiological changes further supported a transient stress state during early recovery. The suppression of photosynthesis‐ and starch biosynthesis‐related transcription during early recovery may reflect temporary adjustments in metabolic activity during the transition from cold stress to normal growth conditions. This response, coupled with the enrichment of genes associated with lipid catabolism in Cluster R4, is consistent with transient metabolic adjustments during recovery.

The circadian clock acts as a pivotal integrator of environmental signals, and its temporal coordination is frequently compromised under diverse abiotic stresses (Kidokoro et al., 2021; Li, Cao, et al., 2019). Our analysis identified widespread phase shifts and reduced oscillation amplitudes among circadian‐associated transcripts, suggesting that cold stress induces extensive temporal remodeling of circadian‐associated expression patterns in maize seedlings. Whether these transcriptional changes reflect alterations in intrinsic circadian clock function or contribute directly to cold tolerance remains to be determined.

COR‐associated transcriptional changes were interpreted within the established JA perception framework rather than as direct evidence of endogenous JA pathway activation. Endogenous JA‐Ile, generated through plant jasmonate biosynthesis, promotes COI1‐JAZ co‐receptor formation and JAZ degradation, whereas COR is a bacterial phytotoxin that mimics JA‐Ile and is perceived through the same receptor module (Katsir et al., 2008; Sheard et al., 2010; Yan et al., 2018). Thus, COR provides a useful chemical perturbation for probing JA‐associated transcriptional responses but does not itself indicate changes in endogenous JA biosynthesis or JA‐Ile accumulation. Given the established role of the JAZ‐ICE‐CBF/DREB1 module in Arabidopsis cold tolerance (Hu et al., 2013), this pathway provides a mechanistic context for the COR‐associated expression patterns observed here. In our dataset, the enrichment of genes associated with “response to jasmonic acid” and the COR‐dependent expression patterns of hormone‐associated transcriptional modules are consistent with modulation of JA‐related transcriptional responses. Nevertheless, because COI1 dependence, JAZ degradation, endogenous JA‐Ile accumulation, and hormone levels were not directly measured, our results indicate hormone‐associated transcriptional remodeling rather than confirmed pathway activation or hormonal crosstalk.

Beyond these cold‐induced circadian‐associated transcriptional changes, the multi‐layered regulatory framework identified here offers a transcriptional perspective on maize cold tolerance. This framework reflects coordinated changes in stress‐responsive and metabolic transcriptional programs, a phenomenon widely recognized as an important component of plant responses under fluctuating environmental conditions (Huot et al., 2014; Zhang et al., 2020). Within this architecture, network‐associated genes such as *ZmWRKY71* and *ZmHSP28* appear to contribute to maize cold tolerance. WRKY TFs have been increasingly identified as regulators of stress‐responsive transcription and hormone‐associated processes (Yang et al., 2025), while heat shock proteins are known to safeguard cellular integrity and prevent protein aggregation under cold‐induced dehydration (Kollipara et al., 2002; Yan et al., 2006). Consequently, these genes represent experimentally supported candidate components and potential genetic resources for improving maize cold tolerance.

Taken together, this study establishes a temporally resolved framework of the maize cold stress response, integrating cold‐induced circadian‐associated transcriptional changes, COR‐associated hormone‐related expression programs, and metabolic reconfiguration to provide insights into the dynamic organization of maize cold responses and potential targets for genetic improvement.

## MATERIALS AND METHODS

### Plant materials and growth conditions

The maize (*Zea mays* L.) inbred line B73 was utilized for high‐resolution time‐course transcriptomic analysis. For functional validation experiments, the inbred line ND101 served as the WT background for the generation of *ZmWRKY71* and *ZmHSP28* overexpression (OE) transgenic lines. The full‐length coding sequences of *ZmWRKY71* and *ZmHSP28* were individually cloned into the pBECXUN vector under the control of the maize *ubiquitin‐1* promoter. The resulting constructs were introduced into ND101 through *Agrobacterium tumefaciens*‐mediated transformation. All maize seeds were germinated in the dark at 25°C for 3 days and then transplanted into growth pots containing a mixture of 66% soil (Pindstrup substrate, Denmark, 10–30 mm) and 34% vermiculite. Seedlings were grown in a controlled growth chamber with a 16 h/8 h light/dark cycle, a constant temperature of 25°C, and 60% relative humidity. Each pot was planted with four uniform seedlings. All subsequent experiments were conducted when the seedlings reached the 12‐day‐old stage (V2‐V3 developmental stage).

### COR pretreatment and cold stress experimental design

To determine the appropriate COR concentration, 12‐day‐old maize seedlings were sprayed with 2 ml per plant of 0, 0.1, 0.5, or 1 μM COR and maintained at 25°C for 24 h before exposure to 4°C for 3 days. The 0 μM COR treatment received an equivalent volume of the solvent‐containing mock solution. Injured leaf area and ion leakage were measured following cold treatment as described below. Each treatment included 10 biological replicates. Statistical significance was assessed using one‐way ANOVA followed by Tukey's multiple‐comparison test (*P* < 0.05). Based on the combined measurements of injured leaf area and ion leakage, 0.5 μM COR was selected for the subsequent experiments.

Uniform 12‐day‐old maize seedlings were randomly assigned to two groups. The COR treatment group was sprayed with 0.5 μM COR solution at a dosage of 2 ml per plant. Non‐COR‐pretreated (mock) seedlings were sprayed with an equal volume of distilled water containing the same proportion of methanol (the solvent used to dissolve COR) to eliminate potential solvent effects. Pretreatment was carried out at 25°C for 24 h.

Subsequently, the seedlings were categorized into four experimental groups to evaluate the effects of COR under different temperature regimes. The normal control (N) and COR pretreatment (NC) groups were maintained consistently at 25°C throughout the experimental period. In contrast, cold stress was induced in the remaining two groups by transferring the seedlings to a 4°C environment, designated as the cold stress (C) and COR pretreatment followed by cold stress (CC) groups, respectively. The cold stress treatment lasted for 36 h, after which the plants were moved back to 25°C for a 24 h recovery period.

### RNA sequencing

Aerial parts of the seedlings were collected at 14 time points during the cold stress period (0, 3, 6, 9, 12, 15, 18, 21, 24, 36 h) and the recovery period (6, 12, 18, 24 h). At 0 h, samples were collected once for each pretreatment condition because N0 and C0, and NC0 and CC0, represented identical conditions before the temperature treatments were initiated. To ensure sample representativeness, each biological replicate consisted of pooled tissues from six uniform seedlings. Two independent biological replicates were prepared for each treatment at each time point, resulting in a total of 108 samples. Total RNA was extracted using Trizol reagent (Invitrogen), and its purity and integrity were assessed using a NanoDrop 2000 and an Agilent 2100 Bioanalyzer. Sequencing libraries were prepared using the Illumina Standard mRNA‐seq library preparation kit (Vazyme), according to the manufacturer's protocol, and sequenced on a HiSeq platform (Illumina).

### Read mapping, transcript assembly, and gene expression analysis

Raw sequencing reads were processed using SolexaQA (V2.5; Cox et al., 2010) to remove low‐quality reads. The cleaned reads were mapped to the maize B73 reference genome (RefGen_v4; Jiao et al., 2017) using Hisat2‐2.0.4 (Kim et al., 2015) with default parameters. The reference genome and annotation files were obtained from the Ensembl Gramene database (http://ensembl.gramene.org/Zea_mays/Info/Index). Transcript assembly and quantification of gene expression levels were performed using Cufflinks (V2.2.0; Ghosh & Chan, 2016) based on uniquely mapped reads, and expressed as FPKM (Fragments Per Kilobase of transcript per Million mapped reads). To ensure experimental rigor, Pearson correlation coefficients between biological replicates were calculated using R software based on normalized expression levels of log_2_ (FPKM value +1.01).

### Hierarchical clustering and PCA

Hierarchical clustering (HCL) was performed using the HCL method in MeV (V4.9) software (https://sourceforge.net/projects/mev‐tm4/files/) based on the norm values of the genes. PCA was conducted using the prcomp function in R software (V4.3.1) with default settings, using log_2_ (FPKM value + 1.01) transformed and normalized expression values as input. For the comparison of transcriptomic data from different sources, the norm values of the genes in each dataset were calculated separately before integrating the datasets for HCL and expression pattern comparison.

### Identification and analysis of cold‐responsive genes, recovery‐responsive genes, COR‐ responsive genes and core cold‐responsive genes

In this study, all gene sets were identified using Cuffdiff from the Cufflinks suite (Trapnell et al., 2012) to calculate expression differences and their significance across treatments. The threshold for significance was uniformly set at |log_2_ (FC)| >1 and *q*‐value <0.05. CRGs were defined as genes exhibiting significant differential expression between the cold stress (C) and normal control (N) groups at any time point during the 0–36 h cold treatment period. Recovery‐responsive genes (RRGs) were designated as genes that exclusively responded significantly during the recovery phase (6–24 h) relative to normal conditions, while showing no significant differential expression during the preceding 0–36 h cold stress period. We identified COR‐responsive genes (COR‐RGs) as genes showing significant differential modulation during the 0–36 h period in response to COR treatment. These were determined through two parallel comparisons: COR‐pretreated normal conditions versus the normal control (NC versus N), and COR‐pretreated cold stress versus the cold stress control (CC versus C). The subset of genes representing the intersection between the CRGs and COR‐RGs datasets was defined as “core cold‐responsive genes” (core CRGs).

### Clustering analysis of temporal expression profiles

The temporal expression profiles of the 12 986 CRGs were subjected to gene‐wise Norm normalization and clustered using the *K*‐means module in MultiExperiment Viewer (MeV, version 4.9). The number of clusters was evaluated using the Figure of Merit (FOM) module by testing candidate cluster numbers from 1 to 20 with the default parameters. Based on the trend of the FOM curve and the interpretability of the resulting temporal expression patterns, 18 clusters were selected for the final *K*‐means analysis. All other parameters were retained at their default settings. The resulting clusters were designated M1‐M18 according to the direction and onset time of their temporal expression changes.

### Gene functional enrichment analysis and construction of TF‐gene association networks

The GO enrichment analysis was carried out by using the Agrigo2.0 website (http://systemsbiology.cau.edu.cn/agriGOv2/index.php). To construct TF‐gene association networks, we employed the context likelihood of relatedness (CLR) algorithm (Faith et al., 2007). First, mutual information (MI) was used to calculate the expression similarity between TF‐gene pairs using the entropy package in R. The CLR algorithm estimated association strength by comparing the MI of a specific pair against the background MI network distribution. The final CLR score was calculated as *f* (*Z*_*I*_, *Z*_*j*_) = SQRT(*Z*_*I*_^2^ + *Z*_*j*_^2^), where *Z*_*I*_ and *Z*_*j*_ represent the corresponding background‐normalized *z*‐scores (Faith et al., 2007). A threshold of *f* (*Z*_*I*_, *Z*_*j*_) > 4 was applied to identify high‐confidence TF‐gene associations.

### Identification and analysis of circadian genes

Circadian genes were identified using the JTK_CYCLE algorithm within the MetaCycle R package (Wu et al., 2016). The analysis was based on the transcriptomic data from the normal control (N) group over a 0–24 h sampling period (with 3 h intervals). Data normalized by Z‐score transformation were utilized for the analysis of circadian rhythms. Genes with significant rhythmic patterns were filtered using the criterion BH.Q < 0.05. To estimate the circadian relative amplitude parameters, the two biological replicates for the 9 phase groups (time points) of each sample were fitted to the following equation using Prism, version 10 (GraphPad Software Inc., San Diego, CA, USA): *Y* = amplitude × cos [(2 × pi × X/wavelength) + phaseshift] + *a*. The results represent the mean ± SE. By comparing the expression profiles under cold stress and normal conditions, phase shifts and amplitude damping were analyzed to characterize cold‐associated changes in the temporal expression of circadian‐associated genes.

### Phenotyping and statistical analysis of maize cold tolerance at the seedling stage

Overexpression (OE) lines and WT (ND101) seedlings were subjected to 4°C for 5 days, followed by recovery at 25°C for 2 days. The damaged area of the second leaf was measured using ImageJ software. The formula used was: Injured area (%) = A2/A1 × 100; A1 = full area of the second leaf; A2 = injured area of the second leaf. For ion leakage assay, aerial tissues of cold‐treated maize seedlings were collected into 15 ml tubes containing 10 ml deionized water. The solution was vacuumed for 30 min, and the initial conductance of the water was measured as S0. After shaking at room temperature for 1 h, the conductance was detected as S1. Then the samples were boiled for 30 min and shaken at room temperature for cooling, followed by detecting the final conductance as S2. The ion leakage was calculated as: Ion leakage (%) = (S1‐S0)/(S2‐S0) × 100.

### RNA extraction and real‐time quantitative polymerase chain reaction (RT‐qPCR)

Aerial tissues from transgenic seedlings were collected, immediately frozen in liquid nitrogen, and stored at −80°C until further processing. For analysis of *ZmARF16* and *ZmHSF24* expression during cold treatment, aerial tissues were collected from ND101 and two independent *ZmWRKY71*‐OE lines at 0, 3, 6, 9, 12, and 24 h after transfer to 4°C. Total RNA was extracted using TRIZOL reagent (Sigma‐Aldrich, Darmstadt, Germany) following the manufacturer's protocol. The concentration and purity of the extracted RNA were assessed using a spectrophotometer, and the RNA was subsequently subjected to reverse transcription using a cDNA synthesis kit (Takara, Kusatsu, Shiga, Japan). RT‐qPCR was performed on a 7500 Fast Real‐Time PCR system (Applied Biosystems, Foster City, CA, USA) using SYBR Green reagent (Accurate Biology, China). The reaction conditions followed the manufacturer's recommended parameters. Specific primers used for the RT‐qPCR assays are listed in Table S2. The transcript levels of target genes were normalized to the internal reference gene *Zmubiquitin1*. Relative gene expression was calculated using the 2^−▵▵Ct^ method (Wang et al., 2024). All experiments were conducted using more than five independent biological replicates, with each biological replicate analyzed in three technical replicates to ensure statistical accuracy.

### Physiological measurements

Leaf chlorophyll status was assessed using a SPAD‐502Plus chlorophyll meter (Konica Minolta, Tokyo, Japan). Measurements were taken at three evenly spaced positions on the second fully expanded leaf, and the mean of the three readings was used as the SPAD value for each plant. Hydrogen peroxide (H_2_O_2_), malondialdehyde (MDA), starch, and soluble sugar contents were determined using the corresponding commercial assay kits (Abbkine, Wuhan, China) according to the manufacturer's instructions. The contents of these compounds were expressed on a fresh‐weight basis.

### Electrophoretic mobility shift assay (EMSA)

Two His‐tagged fusion proteins were expressed in *E. coli* Rosetta (DE3) using the pET‐32a vector, and one MBP‐tagged fusion protein was expressed using the pMAL‐c2X vector. The His‐tagged fusion proteins were purified using nickel‐nitrilotriacetic acid beads (Qiagen, Hilden, Germany), while the MBP‐tagged fusion protein was purified using amylose resin high flow (New England BioLabs (Beijing) Ltd., China). Corresponding mutated probes were generated by replacing the core nucleotides of the predicted cis‐elements. For competition assays, unlabeled wild‐type probes were added at 100‐ or 200‐fold molar excess relative to the biotin‐labeled probes. Biotin‐labeled probes were synthesized by BGI (China). The binding activities of the transcription factors to the target gene promoters were verified using a Chemiluminescent EMSA Kit (Beyotime, Shanghai, China, Cat.#GS009) according to the manufacturer's instructions. The primers and oligonucleotide probes used in this study are listed in Table S2, and the corresponding B73 RefGen_v4 gene model identifiers are provided in Table S3.

### Dual‐luciferase reporter assay

The 2000‐bp promoter sequences of *ZmHSF24*, *ZmARF16*, *ZmBBX26*, *ZmbZIP91*, *ZmHSP28*, and *ZmGA2ox3* were respectively inserted into the pGreenII 0800‐LUC vector, which acted as a reporter. The corresponding B73 RefGen_v4 gene model identifiers and genomic coordinates of the promoter regions used for molecular validation are provided in Table S3. The coding sequences of *ZmWRKY71*, *ZmEREB113*, and *ZmbHLH116* without a stop codon were cloned into the pGreenII 62‐SK vector, which was driven by the *CaMV 35S* promoter and acted as effectors. The empty pGreenII 62‐SK vector served as the negative effector control. The above plasmids were co‐transformed into protoplasts of maize etiolated seedlings and cultured for 12 h under light in a cell culture plate. The detection of LUC activity was performed according to the protocols for the LUC reporter assay system (Promega, Madison, WI, USA). The firefly luciferase/Renilla luciferase (LUC/REN) ratio was used to quantify promoter activity. Five independent transformations were performed for each treatment.

## AUTHOR CONTRIBUTIONS

FY and LD designed and conceived the experiments. TL, WW, and CJ carried out the experiments, analyzed and interpreted the data. TL and FY prepared the manuscript. LD and FY revised the manuscript. All authors have read and agreed to the published version of the manuscript.

## CONFLICTS OF INTEREST

The authors declare no conflict of interest.

## Supporting information


**Dataset S1.** The expression profiles of 25 491 genes expressed in 108 maize samples.

**Dataset S2.** The expression profiles and grouping of CRGs.

**Dataset S3.** All 13 011 edges of the GRN.

**Dataset S4.** The expression profiles and grouping of RRGs.

**Dataset S5.** The expression profiles and rhythmicity of each circadian gene.

**Dataset S6.** The expression profiles of COR‐RGs.

**Dataset S7.** The expression profiles of core CRGs.

**Dataset S8.** Top three hub transcription factors identified from JA, ET, ABA, and SA signaling pathways within Clusters I and III.

**Figure S1.** Determination of the COR concentration used for cold stress experiments and temporal progression of cold‐induced leaf injury in maize seedlings.
**Figure S2.** Correlation between biological replicates of samples for RNA‐seq.
**Figure S3.** Expression patterns of representative cold marker genes during cold and recovery treatments.
**Figure S4.** Comparison of cold‐responsive transcriptional programs at the onset of visible cold injury in untreated and COR‐pretreated maize seedlings.
**Figure S5.** Physiological changes in maize seedlings during cold treatment and subsequent recovery.
**Figure S6.** Expression profiles of circadian‐associated genes during cold stress and subsequent recovery.
**Figure S7.** Overlap between cold‐responsive genes (CRGs) and circadian genes.
**Figure S8.** Overlap between cold‐responsive genes (CRGs) and COR‐responsive genes (COR‐RGs).
**Figure S9.** GO enrichment analysis of Cluster I‐IV genes.
**Figure S10.** Expression profiles of ABA and SA biosynthesis and signal transduction genes in Cluster I and Cluster III.
**Figure S11.** Expression patterns of lipid‐responsive and cell‐cycle‐related genes.
**Figure S12.** Dual‐luciferase reporter assays of selected TF‐target promoter interactions in maize protoplasts.
**Figure S13.** Gene expression profiles of *ZmWRKY71* and *ZmHSP28* across different treatment stages.
**Figure S14.** Construction and verification of *ZmWRKY71‐OE* and *ZmHSP28‐OE* transgenic maize lines.
**Table S1.** Summary of the RNA‐seq data generated from all the samples.
**Table S2.** Primers used in this study.
**Table S3.** Gene identifiers and promoter information used in molecular validation assays.

## Data Availability

The RNA‐seq raw reads that were generated have been deposited in the NCBI Sequence Read Archive (http://www.ncbi.nlm.nih.gov/sra) under accession number PRJNA1429557.
