# Supplementary material for: Deciphering the molecular basis of maize cold tolerance via integrative regulatory network analysis
Source: Plant J. 2026 Oct 29;128(3):e71197. doi: 10.1111/tpj.71197 (PMC13720329; doi:10.1111/tpj.71197)
Supplement: Supplementary file 1 [file TPJ-128-0-s004.docx]

Article type: Research article

**Deciphering the molecular basis of maize cold tolerance via integrative regulatory network analysis**

Tianshuo Li^1^, Wei Wang^1^, Chuang Jiang^1^, Jinkui Cheng^3^, Fei Yi^1^*, Liusheng Duan^1, 2^

^1^State Key Laboratory of Plant Environmental Resilience, Engineering Research Center of Plant Growth Regulator, Ministry of Education &College of Agronomy and Biotechnology, China Agricultural University, No.2 Yuanmingyuan West Road, Hidian, Beijing, China;

^2^College of Agronomy and Biotechnology, China Agricultural University, Beijing, China;

^3^Center for Crop Functional Genomics and Molecular Breeding, China Agricultural University, Beijing, China.

*Corresponding author:

Fei Yi, China Agricultural University

Email: yifei56@cau.edu.cn

**Supplementary Figures**

**Figure S1. Determination of the COR concentration used for cold-stress experiments and temporal progression of cold-induced leaf injury in maize seedlings.**

(a–c) Twelve-day-old maize seedlings were pretreated with 0, 0.1, 0.5, or 1 μM COR and maintained at 25°C for 24 h before exposure to 4°C for 3 days. (a) Representative phenotypes before and after cold treatment. (b) Injured leaf area. (c) Ion leakage. Data are presented as means ± SD (n = 10). Different lowercase letters indicate significant differences among treatments determined by one-way ANOVA followed by Tukey’s multiple-comparison test (*P* < 0.05). Scale bars, 10 cm.

(d) Temporal changes in injured leaf area during the 0-36 h cold-treatment period in cold-stressed seedlings without COR pretreatment (C) and seedlings pretreated with 0.5 μM COR before cold stress (CC). Injured leaf area of the second leaf was quantified using ImageJ and calculated as A2/A1 × 100, where A1 represents the total area of the second leaf and A2 represents the injured area. Data are presented as means ± SD (n = 6). Asterisks indicate significant differences between C and CC at the same time point, as determined by a two-tailed Student’s t-test: ****P* < 0.001.

**Figure S2. Correlation between biological replicates of samples for RNA-seq.** Samples were set up with two biological replicates for all 14 time points. We calculated the Pearson correlation coefficients between biological replicates with the normalized expression levels based on the log_2_ (FPKM value + 1.01) values. The correlation between the biological replicates was high (average *R^2^*=0.95).

**Figure S3. Expression patterns of representative cold marker genes during cold and recovery treatments.**

The line plots show the FPKM-based expression levels of four representative cold marker genes (*ZmDREB1.3*, *ZmMPK1*, *ZmTIP4;3*, and *ZmPRX108*) under normal control (N), cold stress (C), and recovery (RC) conditions. The blue-shaded region indicates the cold treatment period (0-36 h), and the yellow-shaded region represents the recovery period (36-60 h). Black, blue, and green lines correspond to the N, C, and RC treatments, respectively. Each point denotes the mean FPKM value.

**Figure S4. Comparison of cold-responsive transcriptional programs at the onset of visible cold injury in untreated and COR-pretreated maize seedlings.**

(a) Venn diagram showing the overlap between DEGs detected in C9 versus N9 and CC18 versus NC18 comparisons. (b) GO enrichment analysis of the 2,135 DEGs shared by the C9 versus N9 and CC18 versus NC18 comparisons. (c) GO enrichment analysis of the 1,082 DEGs detected exclusively in C9 versus N9. (d) GO enrichment analysis of the 4,256 DEGs detected exclusively in CC18 versus NC18. The dot size represents the number of genes enriched in each GO term, and the color indicates the adjusted *P* value.

**Figure S5. Physiological changes in maize seedlings during cold treatment and subsequent recovery.**

(a) SPAD values during recovery. (b) Ion leakage during recovery. (c) H₂O₂ content during recovery. (d) MDA content during recovery. (e) Starch content during cold treatment and recovery. (f) Soluble sugar content during cold treatment and recovery. For panels (a–d), the x-axis represents cumulative experimental time, and 36 h marks the end of the 36-h cold treatment and the beginning of recovery at 25°C. Thus, 36, 42, 48, 54, and 60 h correspond to 0, 6, 12, 18, and 24 h of recovery, respectively. The orange-shaded background indicates the recovery period at 25°C. In panels (e) and (f), the blue-shaded region indicates the cold-treatment period at 4°C (0–36 h), whereas the orange-shaded region indicates the recovery period at 25°C (36–60 h). C, cold-treated seedlings followed by recovery at 25°C; N, normal-temperature controls maintained at 25°C. Data are presented as means ± SD (n ≥ 4). Different lowercase letters indicate significant differences determined by one-way ANOVA followed by Tukey’s multiple-comparison test (*P* < 0.05). Asterisks indicate significant differences between C and N at the same time point, as determined by a two-tailed Student’s t-test: **P* < 0.05, ***P* < 0.01, and ****P* < 0.001.

**Figure S6. Expression profiles of circadian-associated genes during cold stress and subsequent recovery.**

The heatmap shows the temporal expression patterns of circadian-associated genes in maize seedlings during cold treatment and subsequent recovery. Expression levels are presented as *Z*-scores of FPKM values, with yellow indicating high expression and blue indicating low expression. Samples were collected at multiple time points across both cold stress (0-36 h) and recovery phases.

**Figure S7. Overlap between cold-responsive genes (CRGs) and circadian genes.**

The Venn diagram shows the overlap between CRGs and circadian genes in maize.

**Figure S8. Overlap between cold-responsive genes (CRGs) and COR-responsive genes (COR-RGs).**

The Venn diagram shows the overlap between CRGs and COR-responsive genes identified from the comparisons NC vs. N and CC vs. C. Numbers in red indicate co-responsive genes shared by cold and COR treatments.

**Figure S9. GO enrichment analysis of Cluster I-IV genes.**

The scatter plots show significantly enriched GO terms for genes in Cluster I-IV. The size of each bubble represents the number of genes annotated to the corresponding GO term, and the color indicates the significance level (P. value). The x-axis denotes the Rich Factor, which represents the ratio of the number of enriched genes to the total annotated genes in each GO term. (a-d) correspond to Cluster I, Cluster II, Cluster III, and Cluster IV, respectively.

**Figure S10. Expression profiles of ABA and SA biosynthesis and signal transduction genes in Cluster I and Cluster III.**

The line plots display the normalized FPKM expression levels of genes involved in ABA and SA biosynthesis and signal transduction in Cluster I and Cluster III. The y-axis represents the normalized gene FPKM values across the experimental timeline for four treatment groups: normal control (N), COR pretreatment under normal conditions (NC), cold stress (C) followed by recovery (RC), and COR pretreatment under cold stress (CC) followed by recovery (RCC). Different colored lines correspond to individual genes as indicated in the legends on the right.

**Figure S11. Expression patterns of lipid-responsive and cell-cycle-related genes.**

Line plots display the normalized FPKM expression of genes associated with “response to lipid” in Cluster II (olive lines) and cell-cycle-related genes from Cluster IV (purple lines). Thin lines denote individual genes; thick lines indicate the LOESS-smoothed mean trend within each set.

**Figure S12. Dual-luciferase reporter assays of selected TF-target promoter interactions in maize protoplasts.**

Promoter fragments of *ZmHSF24*, *ZmARF16*, *ZmBBX26*, *ZmbZIP91*, *ZmHSP28*, and *ZmGA2ox3* were fused to the firefly luciferase reporter and co-transfected with effector constructs expressing ZmWRKY71, ZmEREB113, or ZmbHLH116, as indicated. The empty effector vector was used as the control. Promoter activity is expressed as the ratio of firefly luciferase to Renilla luciferase activity (LUC/REN). Data are presented as mean ± SD (n = 5). Statistical significance was determined using a two-tailed Student’s t-test. ***P* < 0.01; ****P* < 0.001.

**Figure S13. Gene expression profiles of *ZmWRKY71* and *ZmHSP28* across different treatment stages.**

The line plots illustrate the normalized FPKM expression levels of *ZmWRKY71* (top) and *ZmHSP28* (bottom) in maize seedlings under four treatment conditions. The y-axis represents normalized gene FPKM values. The x-axis indicates various treatment time points: N, normal control; NC, 0.5 µM COR pretreatment under normal condition; C, cold stress (4°C) and subsequent recovery (RC); CC, 0.5 µM COR pretreatment under cold stress and subsequent recovery (RCC).

**Figure S14. Construction and verification of *ZmWRKY71-OE* and *ZmHSP28-OE* transgenic maize lines.**

(a, b) Schematic diagrams of the pBECXUN::*ZmWRKY71* (a) and pBECXUN::*ZmHSP28* (b) overexpression vector, in which *ZmWRKY71* and *ZmHSP28* are driven by the maize *ubiquitin-1* (*Ubi1*) promoter and tagged with *Myc*. The vector contains a Bar resistance cassette and KanR marker. (c, d) RT-qPCR analysis of relative *ZmWRKY71* (c) and *ZmHSP28* (d) expression levels in OE lines respectively. (e, f) Relative expression of *ZmARF16* (e) and *ZmHSF24* (f) in ND101 and two independent *ZmWRKY71*-OE lines at 0, 3, 6, 9, 12, and 24 h after transfer to 4℃. All expression levels were normalized to that of *Zmubiquitin1*. Error bars represent ± SD (n ≥ 5). Statistical significance was determined using a two-tailed Student’s t-test. **P* < 0.05, ***P* < 0.01, and ****P* < 0.001.

**Supplementary Tables**

| **Table S1. Summary of the RNA-seq data generated from all the samples** | | | | | |  |
| --- | --- | --- | --- | --- | --- | --- |
| **Sample ID** | **Raw reads** | **Mapped reads** | | **Uniq mapped reads** | | **Overall alignment  rate (%)** |
|  |  | **Number** | **%mapped** | **Number** | **%mapped** |  |
| N0-1 | 69605754 | 66812635 | 95.99 | 53081805 | 76.26 | 95.99 |
| N0-2 | 77270802 | 74334887 | 96.20 | 58994089 | 76.35 | 96.20 |
| NC0-1 | 44212762 | 42854190 | 96.93 | 35439632 | 80.16 | 96.93 |
| NC0-2 | 69914586 | 67480315 | 96.52 | 54731356 | 78.28 | 96.52 |
| C3-1 | 42329914 | 40880410 | 96.58 | 33922808 | 80.14 | 96.58 |
| C3-2 | 38969180 | 37624166 | 96.55 | 31273166 | 80.25 | 96.55 |
| N3-1 | 77015960 | 74368378 | 96.56 | 60376162 | 78.39 | 96.56 |
| N3-2 | 40242588 | 39112486 | 97.19 | 32880100 | 81.7 | 97.19 |
| CC3-1 | 42368040 | 41161385 | 97.15 | 34393374 | 81.18 | 97.15 |
| CC3-2 | 43580550 | 42178895 | 96.78 | 34881020 | 80.04 | 96.78 |
| NC3-1 | 65368562 | 63070087 | 96.48 | 50666707 | 77.51 | 96.48 |
| NC3-2 | 75261142 | 72633830 | 96.51 | 58332645 | 77.51 | 96.51 |
| C6-1 | 44277976 | 43066573 | 97.26 | 35938390 | 81.17 | 97.26 |
| C6-2 | 39371410 | 38145241 | 96.89 | 31763265 | 80.68 | 96.89 |
| N6-1 | 72997822 | 70474902 | 96.54 | 56839779 | 77.87 | 96.54 |
| N6-2 | 72266722 | 69950051 | 96.79 | 56942727 | 78.8 | 96.79 |
| CC6-1 | 45518364 | 44115198 | 96.92 | 36475213 | 80.13 | 96.92 |
| CC6-2 | 46607772 | 44737611 | 95.99 | 35876489 | 76.98 | 95.99 |
| NC6-1 | 86078178 | 83168770 | 96.62 | 67145598 | 78.01 | 96.62 |
| NC6-2 | 38290224 | 37167344 | 97.07 | 30904143 | 80.71 | 97.07 |
| C9-1 | 38112752 | 36858483 | 96.71 | 30061308 | 78.87 | 96.71 |
| C9-2 | 45574788 | 44109614 | 96.79 | 34430786 | 75.55 | 96.79 |
| N9-1 | 41985740 | 40348809 | 96.10 | 32821670 | 78.17 | 96.10 |
| N9-2 | 41819942 | 40198059 | 96.12 | 32645586 | 78.06 | 96.12 |
| CC9-1 | 43416800 | 42044042 | 96.84 | 34799776 | 80.15 | 96.84 |
| CC9-2 | 44134548 | 42707724 | 96.77 | 35317352 | 80.02 | 96.77 |
| NC9-1 | 38241262 | 36773517 | 96.16 | 29793443 | 77.91 | 96.16 |
| NC9-2 | 47413636 | 45973132 | 96.96 | 36678088 | 77.36 | 96.96 |
| C12-1 | 43791894 | 42206490 | 96.38 | 34623229 | 79.06 | 96.38 |
| C12-2 | 52117852 | 50188463 | 96.30 | 40996453 | 78.66 | 96.30 |
| N12-1 | 40662246 | 39465567 | 97.06 | 31967339 | 78.62 | 97.06 |
| N12-2 | 41074500 | 39510594 | 96.19 | 32313665 | 78.67 | 96.19 |
| CC12-1 | 44149980 | 42321242 | 95.86 | 33921433 | 76.83 | 95.86 |
| CC12-2 | 45520118 | 43752874 | 96.12 | 35461152 | 77.9 | 96.12 |
| NC12-1 | 40680702 | 38524019 | 94.70 | 30574798 | 75.16 | 94.70 |
| NC12-2 | 45006128 | 42572127 | 94.59 | 33741203 | 74.97 | 94.59 |
| C15-1 | 42819042 | 41504615 | 96.93 | 34392570 | 80.32 | 96.93 |
| C15-2 | 45614492 | 44232710 | 96.97 | 36659826 | 80.37 | 96.97 |
| N15-1 | 46707606 | 44974809 | 96.29 | 37515074 | 80.32 | 96.29 |
| N15-2 | 42113862 | 40554790 | 96.30 | 33803799 | 80.27 | 96.30 |
| CC15-1 | 39526694 | 38374468 | 97.08 | 31883210 | 80.66 | 97.08 |
| CC15-2 | 41475838 | 40227201 | 96.99 | 33241121 | 80.15 | 96.99 |
| NC15-1 | 44597752 | 43350739 | 97.20 | 36609067 | 82.09 | 97.20 |
| NC15-2 | 43664648 | 42353608 | 97.00 | 35362468 | 80.99 | 97.00 |
| C18-1 | 39766394 | 38634370 | 97.15 | 32345476 | 81.34 | 97.15 |
| C18-2 | 40888124 | 39714554 | 97.13 | 33128093 | 81.02 | 97.13 |
| N18-1 | 40030878 | 38829237 | 97.00 | 32685902 | 81.65 | 97.00 |
| N18-2 | 40133796 | 38914865 | 96.96 | 32591778 | 81.21 | 96.96 |
| CC18-1 | 45514212 | 44202320 | 97.12 | 36482607 | 80.16 | 97.12 |
| CC18-2 | 44758522 | 43434450 | 97.04 | 35760994 | 79.9 | 97.04 |
| NC18-1 | 42128602 | 40880555 | 97.04 | 34266954 | 81.34 | 97.04 |
| NC18-2 | 43132660 | 41851524 | 97.03 | 35161219 | 81.52 | 97.03 |
| C21-1 | 38776092 | 37640705 | 97.07 | 31453430 | 81.12 | 97.07 |
| C21-2 | 38892388 | 37734428 | 97.02 | 31391742 | 80.71 | 97.02 |
| N21-1 | 43393748 | 41960872 | 96.70 | 33333746 | 76.82 | 96.70 |
| N21-2 | 44320310 | 43041280 | 97.11 | 35872134 | 80.94 | 97.11 |
| CC21-1 | 46085678 | 44578928 | 96.73 | 35398094 | 76.81 | 96.73 |
| CC21-2 | 38739640 | 37532388 | 96.88 | 29886109 | 77.15 | 96.88 |
| NC21-1 | 43330744 | 41762442 | 96.38 | 33988271 | 78.44 | 96.38 |
| NC21-2 | 43212150 | 41678711 | 96.45 | 34273354 | 79.31 | 96.45 |
| C24-1 | 47464512 | 45997411 | 96.91 | 36694406 | 77.31 | 96.91 |
| C24-2 | 42457292 | 41143765 | 96.91 | 32752274 | 77.14 | 96.91 |
| N24-1 | 47193692 | 45599620 | 96.62 | 36033646 | 76.35 | 96.62 |
| N24-2 | 40235066 | 38989342 | 96.90 | 31003477 | 77.06 | 96.90 |
| CC24-1 | 41794948 | 40058464 | 95.85 | 31084126 | 74.37 | 95.85 |
| CC24-2 | 45101540 | 43203171 | 95.79 | 33587011 | 74.47 | 95.79 |
| NC24-1 | 44595850 | 43313682 | 97.12 | 34950613 | 78.37 | 97.12 |
| NC24-2 | 43244944 | 41992254 | 97.10 | 34053358 | 78.75 | 97.10 |
| C36-1 | 46292064 | 44800965 | 96.78 | 35612983 | 76.93 | 96.78 |
| C36-2 | 38074094 | 36980520 | 97.13 | 30704194 | 80.64 | 97.13 |
| N36-1 | 52639558 | 50942225 | 96.78 | 40687480 | 77.29 | 96.78 |
| N36-2 | 51149226 | 49490401 | 96.76 | 40312848 | 78.81 | 96.76 |
| CC36-1 | 46658176 | 45126138 | 96.72 | 36065164 | 77.3 | 96.72 |
| CC36-2 | 45839176 | 44441030 | 96.95 | 35469502 | 77.38 | 96.95 |
| NC36-1 | 40084756 | 38873435 | 96.98 | 31445329 | 78.45 | 96.98 |
| NC36-2 | 43271242 | 41937578 | 96.92 | 33933049 | 78.42 | 96.92 |
| RC6-1 | 43134718 | 41055470 | 95.18 | 33223254 | 77.02 | 95.18 |
| RC6-2 | 45083330 | 42903094 | 95.16 | 34645975 | 76.85 | 95.16 |
| N42-1 | 45021904 | 43633240 | 96.92 | 35637165 | 79.16 | 96.92 |
| N42-2 | 42476880 | 41206629 | 97.01 | 33704440 | 79.35 | 97.01 |
| RCC6-1 | 51757000 | 49508867 | 95.66 | 38593643 | 74.57 | 95.66 |
| RCC6-2 | 43431770 | 41815091 | 96.28 | 33581994 | 77.32 | 96.28 |
| NC42-1 | 41269314 | 39941664 | 96.78 | 32339010 | 78.36 | 96.78 |
| NC42-2 | 49311978 | 47781753 | 96.90 | 38912695 | 78.91 | 96.90 |
| RC12-1 | 43503120 | 42020649 | 96.59 | 34637571 | 79.62 | 96.59 |
| RC12-2 | 44746174 | 43127383 | 96.38 | 35051654 | 78.33 | 96.38 |
| N48-1 | 40654308 | 39474147 | 97.10 | 31698188 | 77.97 | 97.10 |
| N48-2 | 49230522 | 47739525 | 96.97 | 38649257 | 78.51 | 96.97 |
| RCC12-1 | 53261224 | 51335802 | 96.38 | 39865465 | 74.85 | 96.38 |
| RCC12-2 | 51080242 | 49374429 | 96.66 | 38686977 | 75.74 | 96.66 |
| NC48-1 | 48354900 | 46508987 | 96.18 | 36851239 | 76.21 | 96.18 |
| NC48-2 | 41005652 | 39475448 | 96.27 | 32210102 | 78.55 | 96.27 |
| RC18-1 | 48479156 | 46819279 | 96.58 | 36921365 | 76.16 | 96.58 |
| RC18-2 | 42929194 | 41319235 | 96.25 | 33073465 | 77.04 | 96.25 |
| N54-1 | 40531802 | 38992107 | 96.20 | 31697561 | 78.2 | 96.20 |
| N54-2 | 43893426 | 42145759 | 96.02 | 33785247 | 76.97 | 96.02 |
| RCC18-1 | 42669580 | 41318985 | 96.83 | 32814103 | 76.9 | 96.83 |
| RCC18-2 | 52673748 | 50915200 | 96.66 | 40008116 | 75.95 | 96.66 |
| NC54-1 | 43523182 | 41923633 | 96.32 | 33383779 | 76.7 | 96.32 |
| NC54-2 | 39780078 | 38538709 | 96.88 | 31744947 | 79.8 | 96.88 |
| RC24-1 | 47100702 | 45559268 | 96.73 | 36051824 | 76.54 | 96.73 |
| RC24-2 | 43375280 | 41913496 | 96.63 | 33883664 | 78.12 | 96.63 |
| N60-1 | 43557588 | 42268325 | 97.04 | 35660397 | 81.87 | 97.04 |
| N60-2 | 44064720 | 42523510 | 96.50 | 35141483 | 79.75 | 96.50 |
| RCC24-1 | 54764846 | 52220006 | 95.35 | 41247013 | 75.32 | 95.35 |
| RCC24-2 | 48123616 | 45712990 | 94.99 | 35333779 | 73.42 | 94.99 |
| NC60-1 | 40680472 | 39428422 | 96.92 | 33147396 | 81.48 | 96.92 |
| NC60-2 | 47500350 | 45869688 | 96.57 | 37536742 | 79.02 | 96.57 |

| **Table S2. Primers used in this study** | |
| --- | --- |
| **Primer name** | **Sequence (5'to3')** |
| Zmubiquitin1-RT-F | CTGGTGCCCTCTCTCCATATGG |
| Zmubiquitin1-RT-R | CAACACTGACACGACTCATGACA |
| HSP28-RT-F | CATGAACGGCGAAGGGTG |
| HSP28-RT-R | GCAGCTTCTTGACGGTGAC |
| WRKY71-RT-F | CTCTACTGCAGAAAAGGACTATGG |
| WRKY71-RT-R | GGAACTAGAAGATGGCTTCGC |
| ARF16-RT-F | ATTTTCCTCAAGGCCACGGT |
| ARF16-RT-R | GACTGAGTGGCTGGAGTGTC |
| HSF24-RT-F | AGACGTTCGACCTTGTGGAG |
| HSF24-RT-R | CCCACCTGTCAGGATCAACC |
| WRKY71-ARF16-Bio-F | AGCAGTGTCCGTCAGTAGCAGCGGCGGAGGTGCTCCGCGG |
| WRKY71-ARF16-Bio-R | CCGCGGAGCACCTCCGCCGCTGCTACTGACGGACACTGCT |
| WRKY71-ARF16-MBio-F | AGCAGTGTCAAAAAAAAAAAAAAAAAAAAATGCTCCGCGG |
| WRKY71-ARF16-MBio-R | CCGCGGAGCATTTTTTTTTTTTTTTTTTTTTGACACTGCT |
| WRKY71-ARF16-F | AGCAGTGTCCGTCAGTAGCAGCGGCGGAGGTGCTCCGCGG |
| WRKY71-ARF16-R | CCGCGGAGCACCTCCGCCGCTGCTACTGACGGACACTGCT |
| WRKY71-HSF24-Bio-F | ACCGCCCGCCCTTCTCCCGATTTCACCGACCATTTTTCCC |
| WRKY71-HSF24-Bio-R | GGGAAAAATGGTCGGTGAAATCGGGAGAAGGGCGGGCGGT |
| WRKY71-HSF24-MBio-F | ACCGCCCGCCAAAAAAAAAAAAAAAAAAAACATTTTTCCC |
| WRKY71-HSF24-MBio-R | GGGAAAAATGTTTTTTTTTTTTTTTTTTTTGGCGGGCGGT |
| WRKY71-HSF24-F | ACCGCCCGCCCTTCTCCCGATTTCACCGACCATTTTTCCC |
| WRKY71-HSF24-R | GGGAAAAATGGTCGGTGAAATCGGGAGAAGGGCGGGCGGT |
| EREB113-BBX26-Bio-F | GAGAACCGGCTAACCTCCGGCGGCGAAGAAGTTC |
| EREB113-BBX26-Bio-R | GAACTTCTTCGCCGCCGGAGGTTAGCCGGTTCTC |
| EREB113-BBX26-MBio-F | GAGAACCGGCTAACAAAAAAAAAAAAAAAAGTTC |
| EREB113-BBX26-MBio-R | GAACTTTTTTTTTTTTTTTTGTTAGCCGGTTCTC |
| EREB113-BBX26-F | GAGAACCGGCTAACCTCCGGCGGCGAAGAAGTTC |
| EREB113-BBX26-R | GAACTTCTTCGCCGCCGGAGGTTAGCCGGTTCTC |
| EREB113-bZIP91-Bio-F | AATAGCCTAGCCTCCGCCGCCGTGCCAAGCAGCCGGC |
| EREB113-bZIP91-Bio-R | GCCGGCTGCTTGGCACGGCGGCGGAGGCTAGGCTATT |
| EREB113-bZIP91-MBio-F | AATAGCAAAAAAAAAAAAAAAAAAAAAAAAAGCCGGC |
| EREB113-bZIP91-MBio-R | GCCGGCTTTTTTTTTTTTTTTTTTTTTTTTTGCTATT |
| EREB113-bZIP91-F | AATAGCCTAGCCTCCGCCGCCGTGCCAAGCAGCCGGC |
| EREB113-bZIP91-R | GCCGGCTGCTTGGCACGGCGGCGGAGGCTAGGCTATT |
| bHLH116-HSP28-Bio-1-F | ACACTAGGCTGGACTGCAGGAGCCCAACCGGCCTACGA |
| bHLH116-HSP28-Bio-1-R | TCGTAGGCCGGTTGGGCTCCTGCAGTCCAGCCTAGTGT |
| bHLH116-HSP28-MBio-1-F | ACACTAGGCTGGAAAAAAAAAAAAAAACCGGCCTACGA |
| bHLH116-HSP28-MBio-1-R | TCGTAGGCCGGTTTTTTTTTTTTTTTCCAGCCTAGTGT |
| bHLH116-HSP28-1-F | ACACTAGGCTGGACTGCAGGAGCCCAACCGGCCTACGA |
| bHLH116-HSP28-1-R | TCGTAGGCCGGTTGGGCTCCTGCAGTCCAGCCTAGTGT |
| bHLH116-GA2ox3-Bio-F | CCGCCTTTCTTTCCGACGACACAGCCGCCTAATCTCCGGA |
| bHLH116-GA2ox3-Bio-R | TCCGGAGATTAGGCGGCTGTGTCGTCGGAAAGAAAGGCGG |
| bHLH116-GA2ox3-MBio-F | CCGCCTTTCTAAAAAAAAAAAAAAAAAAAAAATCTCCGGA |
| bHLH116-GA2ox3-MBio-R | TCCGGAGATTTTTTTTTTTTTTTTTTTTAGAAAGGCGG |
| bHLH116-GA2ox3-F | CCGCCTTTCTTTCCGACGACACAGCCGCCTAATCTCCGGA |
| bHLH116-GA2ox3-R | TCCGGAGATTAGGCGGCTGTGTCGTCGGAAAGAAAGGCGG |

| **Table S3. Gene identifiers and promoter information used in molecular validation assays** | | | |
| --- | --- | --- | --- |
| **Gene symbol** | **B73 RefGen_v4 gene model ID** | **Promoter region / genomic coordinates** | **Assay** |
| ZmWRKY71 | Zm00001d006001 | Gene locus, Chr2: 195045688-195052725 | EMSA / dual-LUC / OE |
| ZmEREB113 | Zm00001d010175 | Gene locus, Chr8: 103045015-103050501 | EMSA / dual-LUC |
| ZmbHLH116 | Zm00001d024522 | Gene locus, Chr10: 75942180-75947421 | EMSA / dual-LUC |
| ZmHSF24 | Zm00001d032923 | 2,000-bp promoter, Chr1: 245257733-245259732 | EMSA / dual-LUC / RT-qPCR |
| ZmARF16 | Zm00001d053819 | 2,000-bp promoter, Chr4: 245051594-245053593 | EMSA / dual-LUC / RT-qPCR |
| ZmBBX26 | Zm00001d037735 | 2,000-bp promoter, Chr6: 142434815-142436814 | EMSA / dual-LUC |
| ZmbZIP91 | Zm00001d007042 | 2,000-bp promoter, Chr2: 220713585-220715584 | EMSA / dual-LUC |
| ZmHSP28 | Zm00001d018298 | 2,000-bp promoter, Chr5: 220631212-220633211 | EMSA / dual-LUC / OE |
| ZmGA2ox3 | Zm00001d043411 | 2,000-bp promoter, Chr3: 201140672-201142671 | EMSA / dual-LUC |
